# Mutational Characterization of the Bile Acid Receptor TGR5 in Primary Sclerosing Cholangitis

**DOI:** 10.1371/journal.pone.0012403

**Published:** 2010-08-25

**Authors:** Johannes R. Hov, Verena Keitel, Jon K. Laerdahl, Lina Spomer, Eva Ellinghaus, Abdou ElSharawy, Espen Melum, Kirsten M. Boberg, Thomas Manke, Tobias Balschun, Christoph Schramm, Annika Bergquist, Tobias Weismüller, Daniel Gotthardt, Christian Rust, Liesbet Henckaerts, Clive M. Onnie, Rinse K. Weersma, Martina Sterneck, Andreas Teufel, Heiko Runz, Adolf Stiehl, Cyriel Y. Ponsioen, Cisca Wijmenga, Morten H. Vatn, for the IBSEN study group, Pieter C. F. Stokkers, Severine Vermeire, Christopher G. Mathew, Benedicte A. Lie, Ulrich Beuers, Michael P. Manns, Stefan Schreiber, Erik Schrumpf, Dieter Häussinger, Andre Franke, Tom H. Karlsen

**Affiliations:** 1 Norwegian PSC Research Center, Clinic for Specialized Medicine and Surgery, Oslo University Hospital Rikshospitalet, Oslo, Norway; 2 Research Institute for Internal Medicine, Oslo University Hospital Rikshospitalet, Oslo, Norway; 3 Institute of Clinical Medicine, University of Oslo, Oslo, Norway; 4 Clinic for Gastroenterology, Hepatology and Infectiology, Heinrich-Heine-University, Düsseldorf, Germany; 5 Centre for Molecular Biology and Neuroscience and Institute of Medical Microbiology, Oslo University Hospital Rikshospitalet, Oslo, Norway; 6 Institute of Clinical Molecular Biology, Christian-Albrechts University, Kiel, Germany; 7 First Department of Medicine, University Hospital Schleswig-Holstein, Kiel, Germany; 8 Max Planck Institute for Molecular Genetics, Berlin, Germany; 9 1st Department of Medicine, University Medical Center Hamburg-Eppendorf, Hamburg, Germany; 10 Department of Gastroenterology and Hepatology, Karolinska University Hospital Huddinge, Stockholm, Sweden; 11 Department of Gastroenterology, Hepatology and Endocrinology, Hannover Medical School, Hannover, Germany; 12 Integrated Research and Treatment Center - Transplantation (IFB-Tx), Hannover Medical School, Hannover, Germany; 13 Department of Medicine, University Hospital of Heidelberg, Heidelberg, Germany; 14 Department of Medicine II, Grosshadern, University of Munich, Munich, Germany; 15 Department of Gastroenterology, University Hospital Gasthuisberg, Leuven, Belgium; 16 Department of Medical and Molecular Genetics, King's College London School of Medicine, London, United Kingdom; 17 Department of Gastroenterology and Hepatology, University Medical Center Groningen and University of Groningen, Groningen, The Netherlands; 18 1st Department of Medicine, University of Mainz, Mainz, Germany; 19 Department of Human Genetics, University Hospital of Heidelberg, Heidelberg, Germany; 20 Department of Gastroenterology and Hepatology, Academic Medical Center, University of Amsterdam, Amsterdam, The Netherlands; 21 Department of Genetics, University Medical Center Groningen and University of Groningen, Groningen, The Netherlands; 22 Institute of Clinical Epidemiology and Molecular Biology (Epigen), Akershus University Hospital, Lørenskog, Norway; 23 Clinic for Specialized Medicine and Surgery, Oslo University Hospital Rikshospitalet, Oslo, Norway; 24 Institute of Immunology, Oslo University Hospital Rikshospitalet, Oslo, Norway; Leiden University Medical Center, The Netherlands

## Abstract

**Background:**

TGR5, the G protein-coupled bile acid receptor 1 (GPBAR1), has been linked to inflammatory pathways as well as bile homeostasis, and could therefore be involved in primary sclerosing cholangitis (PSC) a chronic inflammatory bile duct disease. We aimed to extensively investigate *TGR5* sequence variation in PSC, as well as functionally characterize detected variants.

**Methodology/Principal Findings:**

Complete resequencing of *TGR5* was performed in 267 PSC patients and 274 healthy controls. Six nonsynonymous mutations were identified in addition to 16 other novel single-nucleotide polymorphisms. To investigate the impact from the nonsynonymous variants on TGR5, we created a receptor model, and introduced mutated *TGR5* constructs into human epithelial cell lines. By using confocal microscopy, flow cytometry and a cAMP-sensitive luciferase assay, five of the nonsynonymous mutations (W83R, V178M, A217P, S272G and Q296X) were found to reduce or abolish TGR5 function. Fine-mapping of the previously reported PSC and UC associated locus at chromosome *2q35* in large patient panels revealed an overall association between the *TGR5* single-nucleotide polymorphism rs11554825 and PSC (odds ratio  = 1.14, 95% confidence interval: 1.03–1.26, p = 0.010) and UC (odds ratio  = 1.19, 95% confidence interval 1.11–1.27, p = 8.5×10^−7^), but strong linkage disequilibrium precluded demarcation of *TGR5* from neighboring genes.

**Conclusions/Significance:**

Resequencing of *TGR5* along with functional investigations of novel variants provided unique insight into an important candidate gene for several inflammatory and metabolic conditions. While significant *TGR5* associations were detected in both UC and PSC, further studies are needed to conclusively define the role of *TGR5* variation in these diseases.

## Introduction

TGR5, the G protein-coupled bile acid receptor 1 (GPBAR1), was recently identified as the first plasma membrane-bound bile acid receptor [1], [2]. TGR5 is strongly expressed in monocytes and macrophages, and the receptor has been shown to inhibit the release of inflammatory cytokines from activated macrophages [2], [3]. A role in bile homeostasis and metabolic regulation is suggested by *TGR5* knockout mice, which are resistant to gallstones and obesity [4]–[7]. In the hepatobiliary system, TGR5 protein expression has been demonstrated in rodent Kupffer cells, liver sinusoidal endothelium and biliary epithelium [3], [8]. Investigations in humans have so far been limited to the gallbladder, where TGR5 is co-localized with the cystic fibrosis transmembrane conductance regulator (CFTR) [9]. Stimulation of TGR5 in gallbladder cells activates CFTR [9], suggesting that the secretory functions of cholangiocytes may be regulated by this interaction.

Given the bile acid specificity and involvement in inflammatory pathways, TGR5 is a plausible candidate for involvement in hepatobiliary diseases. Primary sclerosing cholangitis (PSC) is a chronic inflammatory condition of the intra- and extrahepatic bile ducts with a prevalence of approximately 10 per 100,000 in Western countries [10], [11]. PSC is strongly linked to inflammatory bowel disease, which affects up to 80% of the patients [12], most often classified as ulcerative colitis (UC), a chronic inflammatory disease of the colonic mucosa [13]. The etiology of PSC and the link to intestinal inflammation is poorly understood [14], but a role of genetic factors in the pathogenesis is likely [15]. TGR5 function has so far not been investigated in PSC, but given the interaction with CFTR, it is interesting that cystic fibrosis (caused by *CFTR* mutations) may involve liver disease, often resembling PSC [16]. Intriguingly, induction of colitis in *Cftr* knockout mice leads to bile duct injury [17], and reduced CFTR function have been reported in PSC patients [18], [19], even in the absence of *CFTR* mutations [19]. Thus, intestinal inflammation seems to increase vulnerability to biliary injury when CFTR function is impaired, and TGR5 could be speculated to be involved.

Little is known about the details of TGR5 structure and how mutations affect function. Whether sequence variation may confer disease susceptibility is also not known. However, the gene is located at a chromosomal region (*2q35*), close to the single-nucleotide polymorphism (SNP) rs12612347 that we recently found associated with both UC [replication OR = 1.18 (1.08−1.28) p = 2.0×10^−4^] and PSC [in a substudy assessing UC findings in PSC, OR = 1.26 (1.06−1.50), p = 0.0088] in genome-wide association studies [20], [21]. Further investigation of this locus has so far not been performed. Given the potential role of TGR5 in bile homeostasis and inflammation, we aimed to make a detailed genetic and functional characterization of TGR5 in PSC, and in parallel assess its possible association with PSC and UC.

## Materials and Methods

### Ethics Statement

This study was approved by the Regional Committee for Medical Research Ethics, South-Eastern Norway, in addition to approval from the ethics committees at all involved centers. Written informed consent was obtained from all participants.

### Subjects

A total of 267 Norwegian PSC patients and 274 healthy controls were included for sequencing of *TGR5* (Table 1). The PSC patients were recruited on admission to Oslo University Hospital Rikshospitalet, while healthy controls were randomly selected from the Norwegian Bone Marrow Donor Registry (NORDONOR). The diagnosis of PSC was based on standard clinical, biochemical, histological and cholangiographic criteria [22]. The diagnosis of inflammatory bowel disease was based on clinical, radiological, histological, and endoscopic (i.e. type and distribution of lesions) criteria [23].

**Table 1:** Characteristics of resequenced individuals.

	PSC (n = 267)	Healthy controls (n = 274)
Age at diagnosis, median (range)	33 (12−73)	32* (18−46)
Male sex, n (%)	196 (73)	157 (57)
Inflammatory bowel disease, n (%)	214 (80)	-
Cholangiocarcinoma, n (%)	27 (10)	-
Liver transplanted or deceased, n (%)	130 (49)	-
Transplantation as primary endpoint, n (%)	87 (33)	-
Observation time to transplantation or death (years), median (range)	8 (0−32)	-

*Age at sampling.

A total of 1109 PSC patients (69% male, 73% with concomitant inflammatory bowel disease), 2761 UC patients (47% male) and 4697 healthy controls were available for genotyping (including the sequenced individuals). These came from Norway and Sweden (panel 1), Belgium and the Netherlands (panel 2), Germany (panel 3) and the United Kingdom (panel 4). Details are described in Materials and Methods S1, including the extent of overlap between the patient panels in the present study and those used in the previous genome-wide association studies. For fine-mapping, a total of 285 Norwegian PSC patients, 882 German and British patients with UC and 2496 healthy controls (subsets of panel 1, 3 and 4) were genotyped.

### TGR5 Resequencing

**Figure 1:**
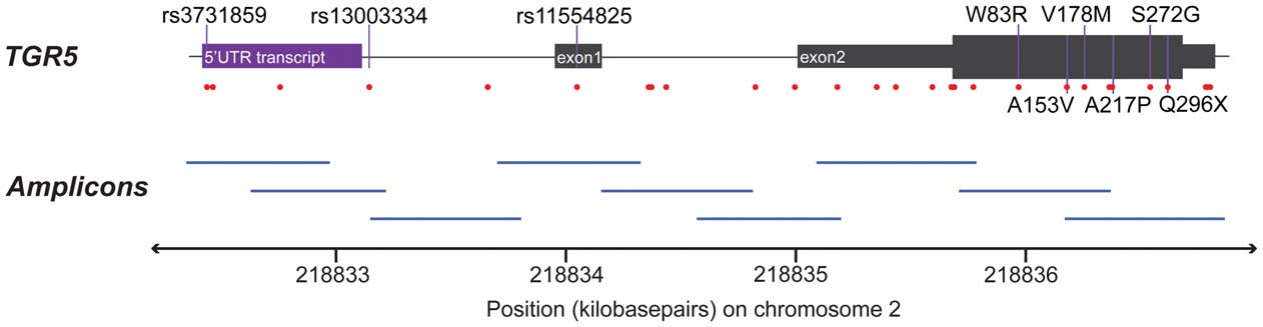
Resequencing of TGR5. The sequenced region of *TGR5,* covering chromosome 2 positions 218,832,394–218,836,917 (NCBI build 36), which includes two exons and the 5′untranslated region (5′UTR), where an alternative transcript has been reported. The coding part of *TGR5* is entirely in exon 2 (thick section). Identified single-nucleotide polymorphisms (SNPs, Table 2) are represented by red dots, and the common SNPs and nonsynonymous mutations are named. The nine amplicons used for sequencing is illustrated below, covering a total of 4524 basepairs non-overlapping sequence with an average amplicon overlap of 174 basepairs.

In order to include all *TGR5* transcript variants so far reported, the entire *TGR5* and 1,589 basepairs of the 5′ region were covered by nine primer pairs (Figure 1 and Table S1). For amplification, a standard touchdown PCR was applied using Taq Gold (Applied Biosystems). A 25 µL reaction contained 2.5 µL ×10 buffer, 3 µL MgCl_2_, 0.5 µL dNTPs (10 mM), 0.4 µL forward primer (10 mM), 0.4 µL reverse primer (10 mM), 0.15 µL taq, 13.05 µL water and 5 µL DNA input (1 ng/µL). The PCR program was as follows: 95°C for 12 min, (95°C for 30 sec, Tm for 30 sec, 72°C for 30 sec)×16 cycles [td −0.5°C], (95°C for 30 sec, Tm−8°C for 30 sec, 72°C for 30 sec) ×19 cycles, 72°C for 10 min, 10°C for ∞. Sequencing was performed using BigDye™ chemistry (Applied Biosystems) according to standard manufacturer's instructions. All sequences were inspected for SNPs, insertions and deletions in both SeqScape v2.5 (Applied Biosystems) and novoSNP v3.0 [24]. All assigned SNPs were observed in both forward and reverse sequences.

### Genotyping

Based on significant associations in UC and PSC genome-wide association studies [20], [21], a 238 kilobasepairs large genetic region was selected for fine-mapping. A HapMap tagging approach was combined with the addition of associated SNPs from the UC study [20] and hand-picked non-HapMap SNPs. A total of 45 SNPs passed assay design and were genotyped using ligation-based SNPlex® genotyping (Applied Biosystems, Foster City, CA, USA) as described elsewhere [25]–[27]. Rs11554825 was genotyped using a TaqMan® assay (Applied Biosystems). Only SNPs with minor allele frequencies >0.01, genotyping success rate >0.95 and no significant deviation from Hardy-Weinberg equilibrium in the healthy controls (*P*>0.01) were included for association analysis.

### TGR5 Structural Modeling and Sequence Data Collection

Publicly available database resources provided by the Ensembl project (http://www.ensembl.org), the DOE Joint Genome Institute (http://genome.jgi-psf.org), the UniProt Consortium (http://www.uniprot.org), and the NCBI (http://www.ncbi.nlm.nih.gov) were searched for homologs of human TGR5. Multiple sequence alignments (MSAs) were generated with Muscle [28], TCoffee [29], MCoffee [30], and Expresso [31], and the MSAs were viewed and manipulated in Jalview [32]. Structural disorder predictions were generated with DISOPRED2 (http://bioinf.cs.ucl.ac.uk/disopred) [33]. A model for the structurally ordered segments of TGR5 was generated from a template 3EML from the Protein Data Bank (http://www.pdb.org) employing standard homology modeling with SwissModel [34]. 3EML is the experimental structure of human adenosine A2a receptor from Jaakola *et al.* [35].

### Cloning and Mutagenesis of Human TGR5

Human *TGR5* was cloned from liver cDNA as previously described [9] (details in Materials and Methods S1). Three different plasmids were generated: 1) TGR5 only, containing the entire coding sequence including the stop codon (pcDNA3.1+ vector, Invitrogen, Karlsruhe, Germany), 2) TGR5-yellow fluorescent protein (TGR5-YFP), containing the coding sequence with a C-terminal YFP-tag (pEYFP-N1 vector, Clontech, Palo Alto, CA, USA), and 3) FLAG-TGR5-YFP, containing the coding sequence with an N-terminal FLAG-tag and a C-terminal YFP-tag (pEYFP-N1). Mutations were introduced using the Multisite-mutagenesis kit (Stratagene, La Jolla, CA, USA). Successful cloning and mutagenesis was verified by sequencing.

### Immunofluorescence Staining

HEK293 (CRL-1573) and MDCK (CCL-34) cell lines were obtained from American Type Culture Collection (Manassas, VA, USA). Both HEK293 (grown on glass coverslips) and MDCK (grown on filterwells) cells were transiently transfected with TGR5 constructs using LipofectAMINE-2000 (Invitrogen). After 48 h cells were fixed in 100% methanol (−20°C, 5 min), immunostained and analyzed on a Zeiss LSM510META confocal microscope using a multitracking modus. A 63x objective and a scanning resolution of 1024×1024 pixels was used for all samples. The following antibodies were used: M39 (TGR5) 1∶500, Na^+^/K^+^ATPase (Sigma, Taufkirchen, Germany) 1∶100, anti-FLAG-M2 (Sigma) 1∶250. Fluoresceine and Cyanine-3 conjugated secondary antibodies (Dianova, Hamburg, Germany) were diluted 1∶100 and 1∶500, respectively. Nuclei were stained with Hoechst (1∶20.000; Invitrogen).

### Flow Cytometry

In order to quantify protein expression and plasma membrane localization, flow cytometry was performed. HEK293 cells were transiently transfected with the TGR5-YFP or FLAG-TGR5-YFP constructs. After 48 h cells were washed with ice-cold phosphate-buffered saline (PBS), detached by pipetting with 1 ml PBS, centrifuged (2,000 g for 3 min at 4°C), resuspended in PBS, and washed twice by centrifugation and resuspension. For measurement of transfection efficacy and protein expression, cell size (forward scatter), granularity (sideward scatter), and fluorescence were analyzed in 50,000 cells using a FACS-CANTO-II (BD Biosciences, Heidelberg, Germany). Cells with YFP-fluorescence greater than 10^3^ were gated and denoted as TGR5-YFP-transfected cells. TGR5 protein expression was measured as mean fluorescence intensity per transfected cell (using the BD FACSDiva Software), normalized to wild-type.

Plasma membrane expression was quantified by determining the amount of the FLAG-tag on the extracellular surface and normalizing to the total amount of TGR5 (determined by YFP-fluorescence): Anti-FLAG M2 was labeled with PacificBlue conjugated anti-mouse IgG-Fab-fragments using the Zenon PacificBlue Label-Kit (Invitrogen). Cells were incubated 30 min (4°C) with the labeled antibody (1∶250). Afterwards cells were washed twice with ice cold PBS and measured for forward scatter, sideward scatter, YFP- and PacificBlue-fluorescence. The following controls were used: Unlabeled non-transfected cells (no fluorescence); unlabeled FLAG-TGR5-YFP transfected cells (only YFP-fluorescence), labeled FLAG-TGR5-Q296X transfected cells (only PacificBlue-fluorescence). The amount of TGR5 in the plasma membrane was determined as the amount of PacificBlue-YFP-positive cells divided by the total amount of YFP-positive cells. As control, detection of every YFP-positive cell with the anti-FLAG antibody in permeabilized cells was performed: Detached cells were fixed in 4% paraformaldehyde (15 min at room temperature), washed with PBS and permeabilized using 0.1% Triton-X (5 min). Afterwards cells were washed twice, incubated with the labeled anti-FLAG-M2 antibody, washed again and analyzed.

### Measurement of cAMP

Receptor activity was investigated using the TGR5 agonist taurolithocholic acid (TLC) [36]. HEK293 cells were cotransfected with a cAMP-sensitive reporter gene construct (Bayer AG, Leverkusen, Germany) and *TGR5* variants in pcDNA, using LipofectAMINE as described [8]. Control experiments were performed with the empty pcDNA vector. Luciferase assays were carried out with Dual-Luciferase kit (Promega, Madison, WI, USA), and luciferase activity was normalized to transfection efficacy, using cotransfection of a *Renilla* expression vector.

### TGR5 Transcription

Expression data for *TGR5* (*GPBAR1*) in EBV-transformed lymphoblastoid cell lines from the HapMap samples was available from the GENEVAR project [37], and expression in the n = 210 non-related individuals were correlated to SNP genotypes, using linear regression in an in-house web-based tool SNPEXP v1.1 (http://app3.titan.uio.no/biotools/tool.php?app=snpexp).

### Statistical Methods

*In vitro* experiments were performed independently at least four times. Results were expressed as means±standard error of the mean, and analyzed using two-sided t-tests. P<0.05 was considered statistically significant. SNP data were analyzed using Plink v1.06 [38] and Haploview v4.1 [39]. Allele frequencies were compared with chi-square tests or the Cochran-Mantel-Haenszel (CMH) test (across multiple panels). Heterogeneity of the odds ratios (ORs) of the panels was assessed with the Breslow-Day test. In order to control for the effect of rs11554825, logistic regressions were performed using Plink conditioning on rs11554825 with inclusion of study panel as a covariate. Linkage disequilibrium (LD) was calculated in healthy controls.

## Results

### Resequencing and Three-Dimensional Modeling of TGR5

Resequencing of the *TGR5* gene in 267 Norwegian PSC patients and 274 healthy controls revealed a total of 29 SNPs (Table 2 and Figure S1). Twenty-two out of 29 were previously not described in any database (novel), and six of these 22 were nonsynonymous. Four nonsynonymous mutations were found in PSC patients only (W83R, A153V, V178M and S272G), one in both a PSC patient and three healthy controls (A217P), and one in a healthy control only (Q296X). Of non-coding variants, three common SNPs in strong LD were detected, of which rs11554825 was located in the untranslated *TGR5* exon 1, while rs3731859 (r^2^ = 0.99 with rs11554825) and rs13003334 (r^2^ = 0.97 with rs11554825) were located in and near a reported transcript in the 5′ untranslated region (Figure 1).

**Table 2:** Single-nucleotide polymorphisms (SNPs) detected at the *TGR5* locus by complete resequencing of the gene (exons, introns, and untranslated regions) in 267 Norwegian PSC patients and 274 healthy controls.

dbSNP ID/name	Chromosome 2 position*	Alleles	Category	Minor allele count (frequency)	
				PSC	Controls
rs3731859	218,832,467	A/G	5′UTR	245 (0.46)	223 (0.41)
TGR5snp1	218,832,492	C/T	5′UTR	2	1
TGR5snp2	218,832,779	C/T	5′UTR	1	1
rs13003334	218,833,166	A/T	5′UTR	241 (0.45)	222 (0.41)
TGR5snp3	218,833,670	A/G	5′UTR	0	1
rs11554825	218,834,054	T/C	Exon 1 untranslated	246 (0.46)	225 (0.41)
TGR5snp4	218,834,358	G/T	Intron	1	0
TGR5snp5	218,834,372	G/A	Intron	1	9
TGR5snp7	218,834,433	G/T	Intron	11 (0.02)	12 (0.02)
TGR5snp6	218,834,814	A/T	Intron	0	1
rs1567869	218,834,980	T/C	Intron	12 (0.02)	12 (0.02)
TGR5snp8	218,835,162	G/C	Exon2 untranslated†	1	1
TGR5snp9	218,835,335	C/T	Exon2 untranslated†	0	1
TGR5snp10	218,835,416	G/A	Exon2 untranslated†	1	0
TGR5snp11	218,835,569	G/A	Exon2 untranslated†	0	1
rs57621524	218,835,654	C/T	Exon 2 untranslated	1	0
rs56192869	218,835,662	G/A	Exon 2 untranslated	1	0
TGR5snp12	218,835,744	T/C	Exon 2 synonymous	0	1
TGR5snp21	218,835,938	T/C	W83R	1	0
TGR5snp22	218,836,149	C/T	A153V	1	0
TGR5snp13	218,836,223	G/A	V178M	1	0
TGR5snp14	218,836,327	C/T	Exon 2 synonymous	5	4
TGR5snp15	218,836,339	G/C	Exon 2 synonymous	11 (0.02)	12 (0.02)
TGR5snp16	218,836,340	G/C	A217P	1	3
TGR5snp17	218,836,505	A/G	S272G	1	0
TGR5snp18	218,836,577	C/T	Q296X	0	1
TGR5snp19	218,836,740	C/G	Exon 2 untranslated	3	2
rs2292549	218,836,750	T/C	Exon 2 untranslated	12 (0.02)	12 (0.02)
TGR5snp20	218,836,759	C/T	Exon 2 untranslated	11 (0.02)	12 (0.02)

*NCBI build 36.

† Resides in exon 2 as depicted in Figure 1, but shorter variants of exon 2 (which do not include the affected nucleotide) have been reported. Alleles =  major/minor allele defined from positive strand. 5′UTR = 5′ untranslated region.

**Figure 2:**
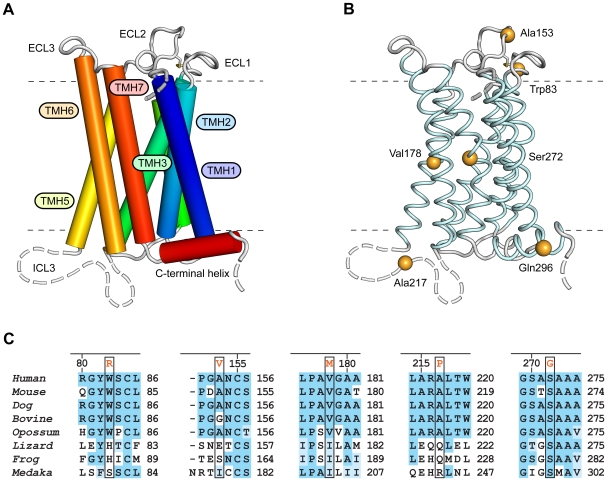
Structure and residue conservation for the family A type G-protein-coupled receptor TGR5. **Panel A** shows the 3D structure of TGR5 as determined by comparative modeling. The receptor comprises seven transmembrane helices (TMH1-7), three extracellular loops (ECL1-3), contributing to ligand binding, and three intracellular loops (ICL1-3) involved in mediating the signal to downstream signaling molecules. ICL3 and the N- and C-terminal segments are structurally flexible and disordered (broken lines). **Panel B** shows the location of the six residues found to be mutated in PSC patients and healthy controls. Evolutionary conservation in sequence segments containing the residues found to be mutated is shown in **Panel C** for a number of mammalian (*Homo sapiens*, *Mus musculus*, *Canis familiaris*, *Bos taurus*, and *Monodelphis domestica*) and other vertebrate species (*Anolis carolinensis*, *Xenopus tropicalis*, and *Oryzias latipes*).

TGR5 is conserved in mammals and other vertebrates. Since a molecular structure of TGR5 was not available, a structural model was generated by comparative modeling in order to predict the role of the nonsynonymous mutations (for details see Results S1). The model shows the seven transmembrane helices standard for G protein-coupled receptors (GPCRs) and a short intracellular C-terminal α-helix (Figure 2A). Residues affected by nonsynonymous mutations were distributed in all parts of the receptor. W83R and A153V were localized in extracellular loops, V178M and S272G in the transmembrane segments and A217P and Q296X in intracellular loops (Figure 2B). These substituted residues were in general conserved in mammals, except Ala153 not conserved in bovine and Ala217 not conserved in guinea-pig (Figure 2C, Figure S2 and Figure S3). Gln296 was not conserved, but Q296X stops the translation of several conserved C-terminal residues.

### Expression and Function of Mutated TGR5

**Figure 3:**
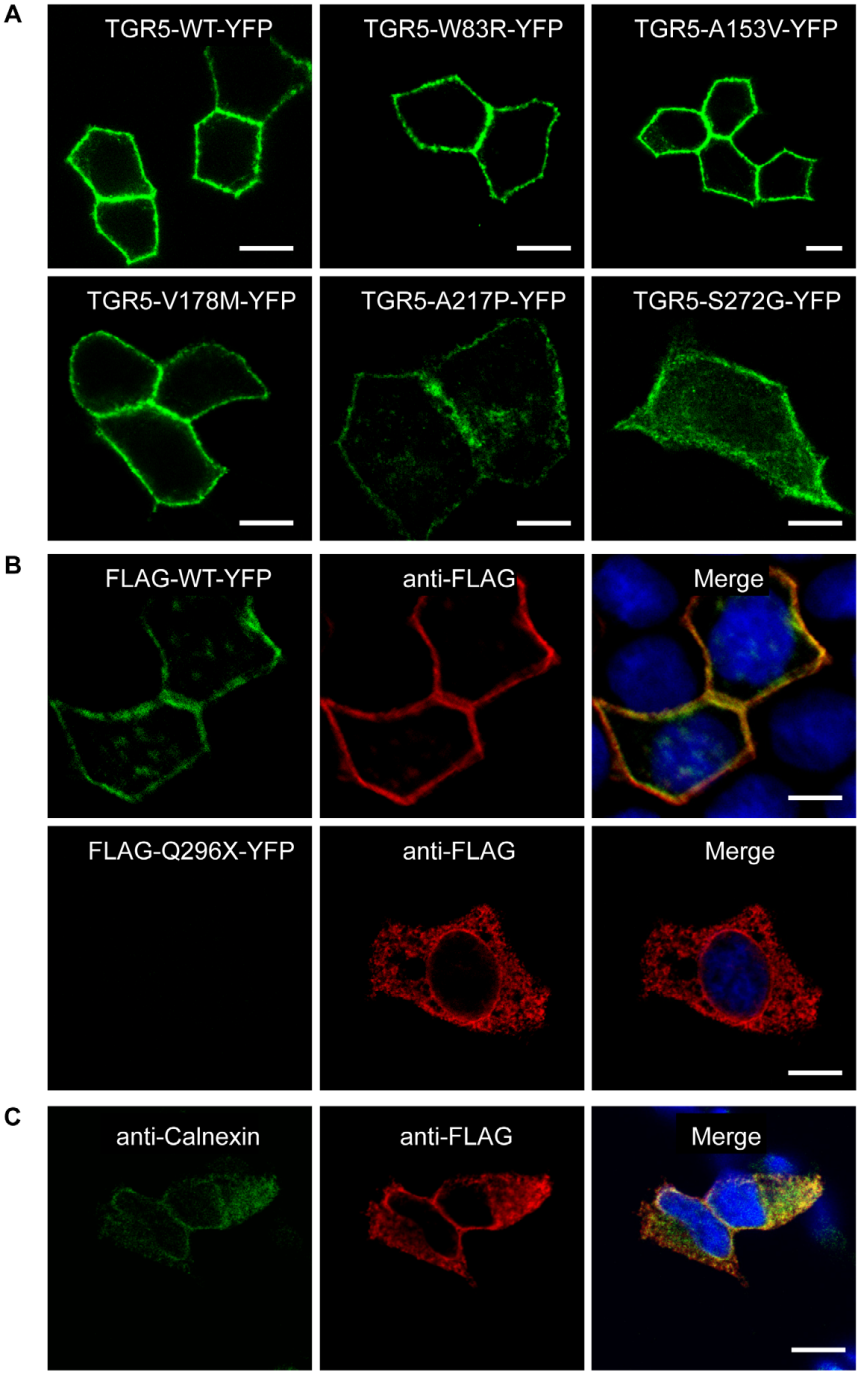
Localization of TGR5 by confocal microscopy. In **Panel A**, polarized MDCK cells were transiently transfected with the different TGR5-YFP variants including wild-type (TGR5-WT-YFP). All variants except TGR5-Q296X-YFP reached the plasma membrane, however TGR5-A217P-YFP and TGR5-S272G-YFP were also present in some intracellular vesicles. In **Panel B**, MDCK cells were transiently transfected with FLAG-TGR5-YFP constructs (wild-type and the Q296X mutant). The FLAG-tag was made visible using an anti-FLAG-M2 antibody (in red) and the yellow coloring in the overlay image demonstrates that the FLAG antibody completely binds to the FLAG-TGR5-YFP wild-type protein both in the plasma membrane as well as in intracellular vesicles (upper row). Introduction of the mutation Q296X leads to a premature stop codon and results in a truncated FLAG-Q296X-YFP protein as demonstrated by the absence of the YFP-fluorescence, but by using the anti-FLAG antibody the truncated protein could be detected (lower row). In **Panel C**, HEK293 cells were transiently transfected with the Q296X mutant of FLAG-TGR5-YFP (for the remaining variants, see Figure S5). The truncated protein, stained with the anti-FLAG antibody, was almost completely retained in the endoplasmatic reticulum, as demonstrated by the colocalization with the endoplasmatic reticulum marker calnexin. Nuclei were stained with Hoechst. Bars = 10 µm.

In order to investigate the effect on receptor localization and expression, the nonsynonymous mutations were introduced into several different constructs and transfected into cell lines. Wild-type TGR5 as well as the W83R, A153V, V178M, A217P and S272G variants all localized to the plasma membrane irrespective of cell line (MDCK or HEK293) or construct (TGR5-YFP, FLAG-TGR5-YFP or TGR5) used, as shown in Figure 3A, Figure S4, Figure S5, Figure S6 and Figure S7. Of particular importance is that TGR5 expressed without tags (Figure S6 and Figure S7) was localized identically to tagged receptors, since tags may alter the localization or function of proteins. When introducing the stop codon (Q296X), the mutated protein was not detected by the TGR5 antibody, which is directed against the C-terminus. Also, when using YFP-tagged constructs, the Q296X mutation resulted in a non-detectable truncated protein without YFP. However, by using an anti-FLAG antibody, FLAG-Q296X-YFP could be detected in the endoplasmic reticulum (ER) as demonstrated by the co-localization with calnexin, a marker protein of the ER (Figure 3B/C).

**Figure 4:**
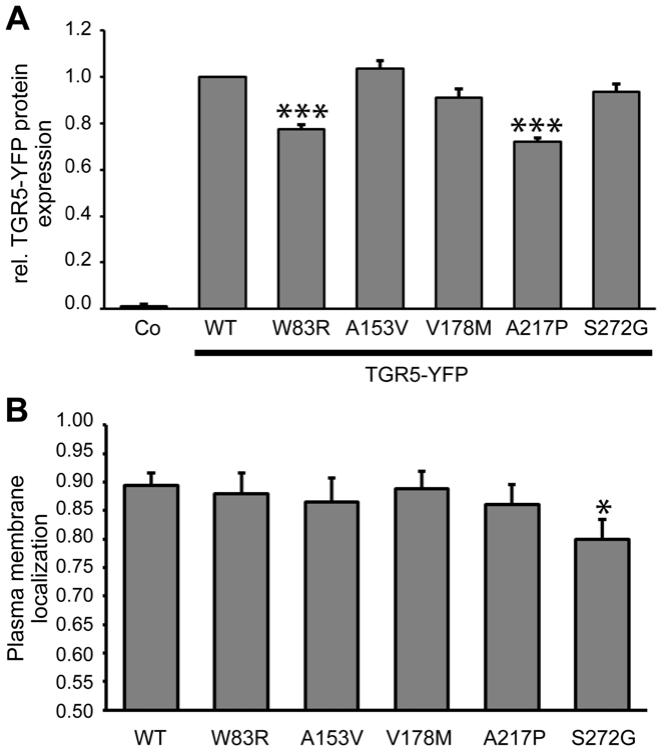
Quantification of expression and localization of TGR5 variants. **Panel A** shows TGR5-YFP protein expression measured by flow cytometry. Mean fluorescence intensity per transfected cell was calculated, and expression of TGR5-WT-YFP (wild-type) was set to 1.0. Untransfected cells (Co) served as controls. The protein expression levels of the W83R (0.77±0.02, n = 6) and A217P (0.72±0.02, n = 6) variants were reduced. Results are shown as mean±standard error of the mean. ***Indicates significant difference (p<0.001) compared with wild-type. **Panel B** shows membrane localization of FLAG-TGR5-YFP variants as determined by flow cytometry. The N-terminal FLAG-tag is localized extracellularly and can be labeled with antibodies only when TGR5 is localized in the plasma membrane. The amount of receptor within the plasma membrane was determined by dividing the amount of FLAG-labeled TGR5 by the total amount of TGR5, as determined by YFP-fluorescence. In HEK293-cells 89±2% (n = 7) of wild-type was located in the plasma membrane, which was similar to W83R, A153V, V178M and A217P. The S272G variant showed significantly reduced plasma membrane localization compared with wild-type (80±3%, n = 7). Results are shown as mean±standard error of the mean. *Indicates significant difference (p<0.05) as compared with wild-type.

Subsequently, the amount of protein produced and the proportion actually localized within the plasma membrane, was quantified using YFP-constructs and flow cytometry analysis in non-permeabilized and permeabilized cells. Protein expression of TGR5-W83R and TGR5-A217P was significantly reduced as compared with wild-type TGR5 (Figure 4A), even though transfection efficacy was similar for all constructs (Figure S8). In HEK293 cells 89% of wild-type TGR5 was located in the plasma membrane, which was similar for the W83R, A153V, V178M and A217P variants, while TGR5-S272G showed significantly reduced plasma membrane localization (80%, p = 0.025, Figure 4B).

**Figure 5:**
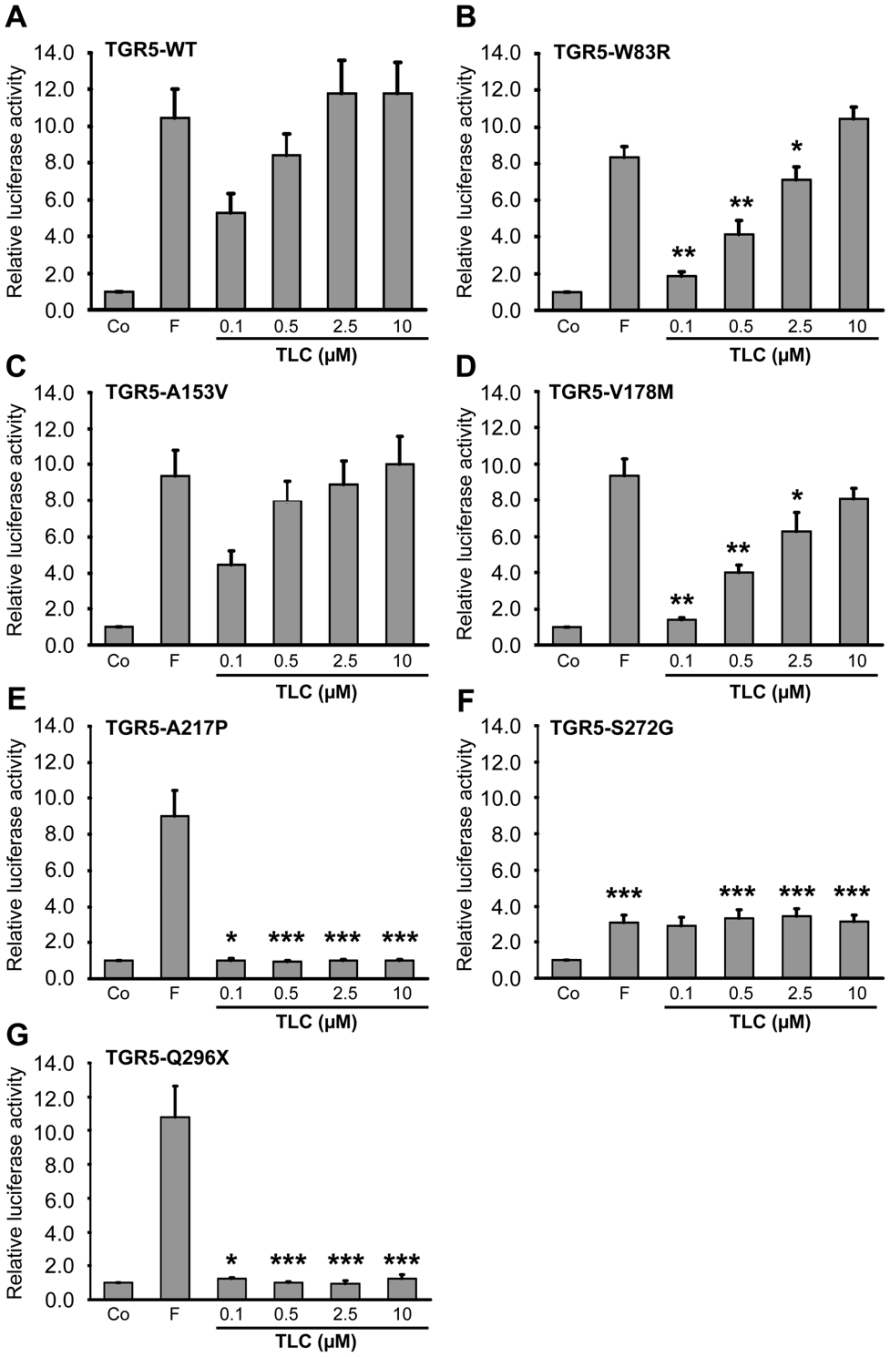
Activation of TGR5 variants by the bile acid taurolithocholic acid (TLC). HEK293 cells were cotransfected with TGR5 and a reporter gene containing five cAMP responsive elements in front of the luciferase gene. Luciferase activity served as a measure of the rise in intracellular cAMP following activation of TGR5. Forskolin (F, 10 µM) stimulated cAMP production independently of TGR5 and served as positive control. Results are expressed as mean±standard error of the mean. *Indicates significant difference (p<0.05) as compared with TGR5 wild-type (TGR5-WT). **p<0.01. ***p<0.001. In **Panel A**, activation of TGR5 wild-type (TGR5-WT) by TLC led to significant rises in luciferase activity already at a concentration of 0.1 µM (n = 14). **Panel B and D** show significantly reduced responses from TGR5-W83R (n = 4) and TGR5-V178M (n = 9) to 0.1, 0.5 and 2.5 µM TLC compared with TGR5-WT, while the responses to forskolin and 10 µM TLC were unaffected. The A153V variant did not affect receptor activity (**Panel C**, n = 12).The A217P (**Panel E**, n = 6) and Q296X (**Panel G**, n = 7) variants completely lost responsiveness to TLC. **Panel F** shows the S272G variant, which exhibited suppressed activation of luciferase by both forskolin and TLC at concentrations of 0.5, 2.5 and 10 µM (n = 11).

TGR5 activity was analyzed using a cAMP-luciferase reporter gene assay following stimulation with TLC, which is the most potent endogenous agonist [36]. Forskolin increases cAMP independently of TGR5 and was used as positive control. Wild-type TGR5 exhibited high activity at 0.5 µM TLC and saturated at 2.5 µM (Figure 5A). W83R and V178M showed a significant reduction in activity at TLC concentrations between 0.1 and 2.5 µM, while activation by forskolin and 10 µM TLC were unchanged (Figure 5B/D). The activity of A153V was similar to wild-type (Figure 5C). The A217P and Q296X variants completely abolished TGR5 responsiveness towards TLC with unaffected activation by forskolin (Figure 5E/G). Introduction of S272G led to significant reduction in luciferase activity following stimulation with both forskolin and TLC (Figure 5F).

### Genetic Associations at Chromosome 2q35

**Figure 6:**
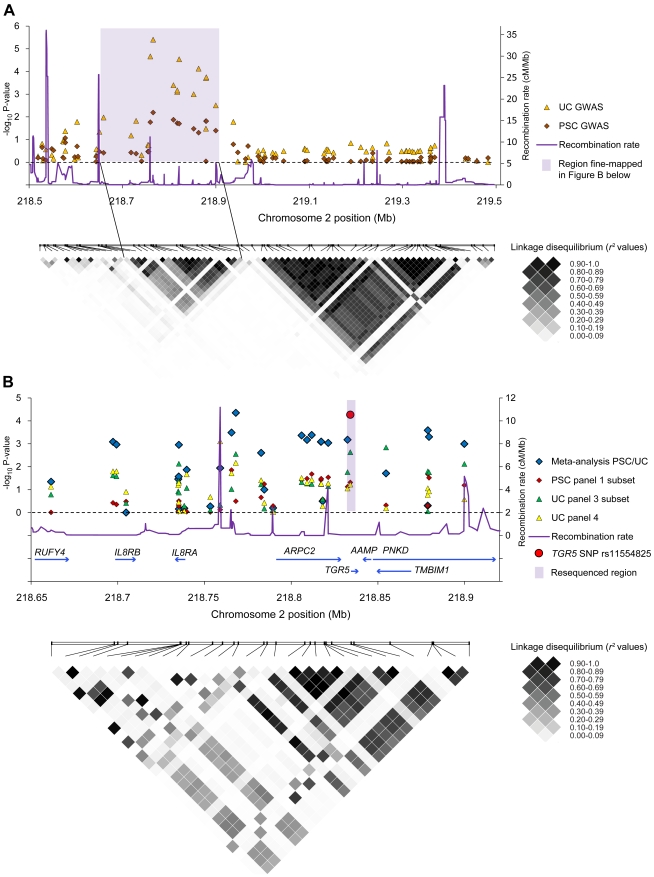
Genetic analyses at chromosome *2q35*. **Panel A** shows results from the association analysis (negative log_10_-transformed p-values plotted on the vertical axis) of individual single-nucleotide polymorphisms (SNPs) at chromosome *2q35* in data sets from previous genome-wide association studies (GWAS) in primary sclerosing cholangitis (285 patients and 298 healthy controls) and ulcerative colitis (1167 patients and 777 healthy controls) [20], [21]. A linkage disequilibrium (LD) plot below shows pairwise LD (*r* ^2^) between the SNPs, calculated in the healthy controls, where increasing *r* ^2^ values correspond to increasing LD. The shaded area covers the peak of associated SNPs corresponding to a region with strong LD. **Panel B** shows fine-mapping of the shaded region in **Panel A** performed in three patient panels (details of the analysis are shown in Results S1). The individuals in the PSC panel 1 subset (285 patients and 296 healthy controls) completely overlapped with the PSC GWAS in panel A, while the individuals in the UC panel 3 subset (521 patients, 1096 controls) and UC panel 4 (361 patients, 1104 controls) were independent from the UC GWAS in panel A. In addition to association analyses in the individual study panels, a PSC-UC meta-analysis of all fine-mapped patients (n = 1167) and healthy controls (n = 2496) was performed. The fine-mapped region was characterized by strong LD, as shown in the lower plot, but a recombination hot-spot was present between the *IL8RA* and *IL8RB* loci and the *TGR5* (*GPBAR1*) locus and neighboring genes (*ARPC2-TGR5-AAMP-TMBIM1-PNKD*). The meta-analysis p-value of the *TGR5* exon 1 SNP rs11554825 is **highlighted in red**, while the shaded area in Panel B shows the resequenced region covering *TGR5*.

In parallel with *TGR5* sequencing, fine-mapping at chromosome *2q35* was performed in PSC and UC. Figure 6A shows overlapping and distinct peaks of statistically significant associations at chromosome *2q35* in SNP data from previous genome-wide association studies in PSC and UC [20], [21]. Further fine-mapping in PSC and UC panels, including a joint PSC-UC meta-analysis of the fine-mapped panels, was subsequently performed in the peak region (Figure 6B). A high degree of LD was revealed, meaning that alleles at TGR5 and several neighboring loci occurred more frequently together than would have been expected by chance (Figure 6B). A recombination hot-spot separated the *IL8RA* and *IL8RB* genes from a LD block encompassing *TGR5* and flanking genes, but further demarcation of a susceptibility variant at chromosome *2q35* was not possible.

Following up on the findings from the *TGR5* sequencing, the only common exonic SNP in the gene was subsequently genotyped in all available study panels. In the combined analysis in PSC (1109 patients and 3593 healthy controls) and UC (2761 patients and 4697 healthy controls), rs11554825 was associated with both PSC (OR = 1.14; 95% confidence interval: 1.03–1.26, p_CMH_ = 0.010) and UC (OR = 1.19; 95% confidence interval: 1.11–1.27, p_CMH_ = 8.5×10^−7^), see Table 3 for allele frequencies. The originally reported intergenic SNP at *2q35* (rs12612347) [20], [21] was associated at a similar level [PSC OR = 1.15 (1.05−1.27), p_CMH_ = 0.0044 and UC OR = 1.19 (1.11−1.28), p_CMH_ = 5.6×10^−7^]. The rs11554825 and rs12612347 SNPs were in strong LD (*r^2^* = 0.73), and logistic regression demonstrated that the effects were not independent. However, when controlling for the effect of rs11554825 (Figure 6B, highlighted in red) in the joint PSC-UC analysis of the fine-mapping panels, three SNPs in LD showed evidence of independent association (considering p<0.05 as the threshold), with the lowest p-value at rs4674271 (p_rs11554825_ = 0.0025, Results S1). These SNPs had minor allele frequencies of 0.04–0.05 (in the healthy controls) and were in LD with other low frequency SNPs throughout the locus (Figure 6B and Results S1). In conclusion, the original reported association at chromosome *2q35* may be caused by rs11554825, but several independent variants could have an effect at this locus.

**Table 3:** Minor allele frequencies and association analysis of *TGR5* SNP rs11554825.

	Panel 1	Panel 2	Panel 3	Panel 4		
	n*	n	n	n	OR (95% CI)	P_CMH_ †
	666/422/298	1095/331/583	1832/356/1519	1104/NA/361		
Healthy controls	0.407	0.412	0.414	0.434		
PSC	0.459	0.440	0.432	NA	1.14 (1.03−1.26)	0.010
UC	0.471	0.429	0.463	0.475	1.19 (1.11−1.27)	8.5×10^−7^

*n: number of healthy controls/PSC patients/UC patients;

† Breslow-Day test yielded no evidence for heterogeneity of odds ratio (P-values 0.52 and 0.40, respectively). CI, confidence interval; OR, odds ratio (compared with healthy controls); NA, not applicable; P_CMH_, p-value as calculated with Cochran-Mantel-Haenszel test across available panels; PSC, primary sclerosing cholangitis; UC, ulcerative colitis.

The effect of rs11554825 on *TGR5* mRNA expression was investigated in a publically available dataset from lymphoblastoid cell-lines. Alleles at the PSC and UC associated rs11554825 were in almost perfect LD with alleles at the 5′untranslated region (5′UTR) rs3731859 (*r* ^2^ = 0.99, Figure 1). There was a weak but significant correlation between expression of *TGR5* and rs3731859 genotypes [*r^2^* (explained variance)  = 0.048, p = 0.0015, Figure S9]. The lowest expression was observed in individuals homozygous for allele G, which was linked to risk allele C at rs11554825.

## Discussion

The dual function of TGR5 in biliary bicarbonate secretion and Kupffer cell inhibition along with findings in previous genome-wide association studies provided a strong rationale for the search for novel genetic variants of *TGR5* in PSC. Extensive resequencing identified several nonsynonymous mutations, and the detected effects on TGR5 structure, expression and function provide valuable insight into the structural biology of the receptor. The statistical associations detected for one exonic SNP in both PSC and UC emphasize the possibility that TGR5 may influence disease susceptibility in these conditions, but further studies are required to exclude a role for neighboring genes.

GPCRs are important mediators of physiological responses, as well as therapeutic targets [40]. It has proven very challenging to resolve experimentally the molecular structure of GPCRs [40]. The structure-function relationship and effects of mutations are therefore of great interest both for TGR5 and GPCRs in general. One example is the A217P mutation, which caused a complete loss of TGR5 activity as measured by the luciferase assay, pointing to a crucial role of the third intracellular loop for TGR5 function. In other GPCRs this loop has been found to be important for interaction with the G protein [41], and interestingly, it is a key target for a new class of GPCR specific agonists and antagonists named pepducins [42]. The residues Trp83 and Val178 are both conserved in mammals, and the mutations W83R and V178M led to significantly reduced TGR5 response to TLC concentrations (0.1–2.5 µM) close to the concentration known to induce half maximal activity of wild-type TGR5 (0.29 µM) [36]. This reduced activity corresponds to a right-shifted dose-response curve, implying either reduced affinity or efficacy of the agonist. For W83R this may be explained by the position of Trp83 in the first extracellular loop, suggesting that the mutation affects ligand binding. However, the accuracy of the receptor model in this part of the molecule was too low to allow detailed studies of possible mechanisms. The reduced activity of the V178M mutated protein could possibly be explained by the localization of Val178 in the fifth transmembrane helix, close to conserved residues involved in propagation of conformational changes from the ligand-binding pocket to the G-protein [43].

The S272G mutation abolished both responsiveness towards TLC (TGR5 dependent) and forskolin (TGR5 independent), suggesting altered interaction of TGR5 with downstream signaling targets. Ser272 is conserved in all vertebrates and positioned in the seventh transmembrane helix, likely to be important for transmitting conformational changes. One possibility is that S272G is an example of a constitutively active mutant, non-responsive to ligands, which simultaneously diminishes the response to forskolin by saturating adenylate cyclase [44]. The A153V mutation appeared to be without functional consequences when using TLC as agonist, but it can not be excluded that mutation of this extracellular residue could affect the response to other endogenous agonists. Although minor changes in TGR5 expression and plasma membrane localization were noted for several mutations, the premature stop codon Q296X was the only mutation with a major impact. By deleting 35 C-terminal residues, it led to an almost complete retention of the receptor in the endoplasmatic reticulum. This indicates that the cytoplasmic tail is required for normal surface expression, in line with observations made with the G protein-coupled angiotensin receptor AT_1a_ [45]. In summary our results show that nonsynonymous mutations may critically affect targeting of TGR5 to the membrane, agonist binding, propagation of conformational changes through the membrane, or intracellular signaling.

The *TGR5* common SNP was statistically associated with both PSC and UC in large European patient panels, but conclusive evidence defining *TGR5* as a disease gene in these conditions could not be established. The main challenge was strong LD spanning the locus, which is a common obstacle to the conclusive identification of disease genes [46]. In addition there was statistical evidence for independently associated risk variants at *2q35*, but the importance and nature of such variants can only be speculated based on the present data. Regarding *TGR5*, further experimental studies along with genetic mapping in other ethnicities will be needed to overcome the limitations in present data. The important notion that nonsynonymous mutations were observed in both PSC patients and healthy controls is not surprising, since only heterozygosity was observed, and genetically complex traits like PSC and UC are caused by an interplay of multiple genetic variants and environmental factors [47], [48]. The mutations were too rare to explain the statistical associations, but yielded an opportunity to investigate the variable effects from naturally occurring genetic variation.

For common variants, the biological effects are often weak and difficult to elucidate, and the correlation between expression levels of *TGR5* and rs11554825 genotypes was not strong, but still in line with recent findings in other diseases [49], and could therefore be speculated to contribute to the associations with PSC and UC. Furthermore, while the effect size was weak in statistical terms (OR), the association may still be important by suggesting an involvement of TGR5 related pathways in PSC and UC [50]. Further exploration of the implicated mechanisms of such an involvement may shed new light on the role of bile acids in intestinal and biliary inflammation, which could ultimately have therapeutic implications. This is particularly relevant for PSC, where no medical treatment has yet been shown to influence progression to liver cirrhosis and liver transplantation [51].

## Supporting Information

Materials and Methods S1 Supporting information about the subjects and the cloning and mutagenesis of human TGR5.
(0.05 MB DOC)

Results S1 Supporting results from the resequencing of TGR5, the sequence collection, the structure modeling and the genetic associations at chromosome *2q35*.
(0.09 MB DOC)

Figure S1 Chromatograms from one heterozygote of each SNP detected through resequencing of TGR5.
(1.14 MB DOC)

Figure S2 Multiple sequence alignment of human TGR5 and orthologs (N-terminal half). Human TGR5 and orthologs from the primates *Pan troglodytes* (chimpanzee), *Pongo pygmaeus* (orangutan), *Macaca mulatta* (macaque), and *Otolemur garnettii* (bushbaby), other mammals such as *Oryctolagus cuniculus* (rabbit), *Cavia porcellus* (guinea pig), *Dipodomys ordii* (kangaroo rat), *Mus musculus* (mouse), *Rattus norvegicus* (rat), *Pteropus vampyrus* (flying fox bat), *Equus caballus* (horse), *Dasypus novemcinctus* (armadillo), *Canis familiaris* (dog), *Bos taurus* (cow), *Tursiops truncatus* (dolphin), the marsupial *Monodelphis domestica* (opossum), the lizard *Anolis carolinensis*, the frogs *Xenopus tropicalis* and *X. laevis*, and the fish *Oryzias latipes* (medaka), *Gasterosteus aculeatus* (three-spined stickleback), *Takifugu rubripes* (fugu), and *Tetraodon nigroviridis*. Residues found to be mutated in humans have been highlighted, and the Cys85-Cys155 disulfide bridge has been indicated (dashed line).
(3.55 MB TIF)

Figure S3 Multiple sequence alignment of TGR5 (C-terminal half) from the same species as in Figure S2. Residues found to be mutated in humans have been highlighted.
(6.29 MB TIF)

Figure S4 Localization of TGR5-YFP variants in HEK293 cells. HEK293 cells were transiently transfected with the different TGR5-YFP variants. An antibody against Na^+^/K^+^-ATPase was used to stain the plasma membrane (shown in red). Nuclei were stained with Hoechst (blue). A. Wildtype TGR5 (WT) was almost completely targeted to the plasma membrane. B. The mutant TGR5-W83R-YFP was also localized in the plasma membrane as demonstrated by the colocalization with the Na^+^/K^+^-ATPase resulting in a yellow coloring in the overlay picture. C. Introduction of A153V into TGR5-YFP did not affect plasma membrane localization. D. TGR5-V178M-YFP was also targeted to the plasma membrane as shown by the colocalization with the Na^+^/K^+^-ATPase fluorescence pattern. E. TGR5-A217P-YFP was also detected in the plasma membrane as demonstrated by the yellow coloring in the superimposed image. F. TGR5-S272G-YFP was both localized in the plasma membrane but also in some intracellular vesicles. Bars = 10 µm.
(5.32 MB TIF)

Figure S5 Localization of FLAG-TGR5-YFP in HEK293 cells. HEK293 cells were transiently transfected with the different FLAG-TGR5-YFP constructs. The FLAG-tag was made visible using an anti-FLAG-M2 antibody (in red). The yellow coloring in the overlay images demonstrate that the FLAG antibody completely binds to the FLAG-TGR5-YFP proteins both in the plasma membrane as well as in intracellular vesicles (A–F). Nuclei were stained with Hoechst. Bars = 10 µm.
(4.35 MB TIF)

Figure S6 Localization of TGR5 in pcDNA in HEK293 cells. HEK293 cells were transiently transfected with the different TGR5 variants (without tags) and stained for TGR5 using the anti-TGR5 (M39) antibody (shown in red). An antibody against Na^+^/K^+^-ATPase was used as a marker for the plasma membrane (shown in green). Nuclei were stained with Hoechst (blue). Wildtype TGR5 (TGR5-WT) was almost completely targeted to the plasma membrane. All TGR5-variants were also localized in the plasma membrane of HEK293 cells as demonstrated by the colocalization with the fluorescence from the Na^+^/K^+^-ATPase antibody.
(4.21 MB TIF)

Figure S7 Localization of TGR5 in pcDNA in MDCK cells. TGR5 variants were transfected into polarized MDCK cells. TGR5 and TGR5 mutants were all detected in the plasma membrane using the anti-TGR5 antibody (M39 in red). Bars = 10 µm.
(1.29 MB TIF)

Figure S8 Transfection efficacy of TGR5-YFP in HEK293 cells. HEK293 cells were transiently transfected with TGR5-YFP. Transfection efficacy was determined by flow cytometry.
(0.10 MB TIF)

Figure S9 TGR5 expression in Epstein-Barr-virus-transformed lymphoblastoid cell lines according to rs3731859 genotypes in all non-related HapMap individuals (n = 210). Expression levels were retrieved from the GENEVAR project. Genotypes and height of expression were significantly correlated (*r* ^2^ = 0.048, p = 0.0015) in a linear regression analysis performed in SNPexp v1.1 (http://app3.titan.uio.no/biotools/tool.php?app=snpexp, which utilizes Plink v1.06).
(0.04 MB TIF)

Table S1 Details of the nine primer pairs used in TGR5 resequencing (M13-primer-sequence underlined).
(0.04 MB DOC)

Note S1 List of members in the IBSEN study group.
(0.03 MB DOC)
