# Supplementary material for: Mutational Characterization of the Bile Acid Receptor TGR5 in Primary Sclerosing Cholangitis
Source: PLoS One. 2010 Aug 25;5(8):e12403. doi: 10.1371/journal.pone.0012403 (PMC2928275; doi:10.1371/journal.pone.0012403)
Supplement: Materials and Methods S1 — Supporting information about the subjects and the cloning and mutagenesis of human TGR5. (0.05 MB DOC) [file pone.0012403.s001.doc]

**SUPPLEMENTARY MATERIALS AND METHODS**

*SUBJECTS* Details on the PSC and UC patient panels are shown below:

| **Panel^A^** | **Origin** | **Healthy controls** | | **PSC** | | | **UC** | |
| --- | --- | --- | --- | --- | --- | --- | --- | --- |
|  |  | **n** | **% male** | **n** | **% male** | **% IBD** | **n** | **% male** |
| Panel 1 | Norway and Sweden | 666 | 65 | 422^B^ | 72 | 81 | 298 | 52 |
| Panel 1 subset | Norway | 296 | 59 | 285 | 74 | 80 | NA | NA |
| Panel 2 | Belgium and the Netherlands | 1095 | 57 | 331^C^ | 65 | 67 | 583^D^ | 50 |
| Panel 3 | Germany | 1832 | 41 | 356 | 69 | 67 | 1519 | 44 |
| Panel 3 subset | Germany | 1096 | 35 | NA | NA | NA | 521 | 46 |
| Panel 4 | United Kingdom | 1104 | 50 | NA | NA | NA | 361 | 48 |

^A^E.g. PSC panel 1 consisted of 422 PSC patients and 666 healthy controls from Norway and Sweden. UC panel 3 subset consisted of 521 UC patients and 1096 healthy controls from Germany. ^B^N=295 from Norway and n=127 from Sweden. ^C^N=142 from Belgium and n=189 from the Netherlands. ^D^All from the Netherlands. IBD, inflammatory bowel disease; NA, not applicable; PSC, primary sclerosing cholangitis; UC, ulcerative colitis.

The PSC patients in panel 1 were recruited on admission to the Medical Department of Rikshospitalet, Oslo University Hospital, Oslo, Norway and Huddinge University Hospital, Stockholm, Sweden. PSC patients in panel 2 were recruited from University Hospital Leuven, Belgium, and the Academic Medical Center, Amsterdam and the University Medical Center, Groningen in the Netherlands. German PSC patients in panel 3 were recruited from Grosshadern University Clinic, Munich, and the University Hospital of Heidelberg, Heidelberg, or through the Northern German biobank Popgen (www.popgen.de) from patients recruited at the University Medical Center Hamburg-Eppendorf, the Hannover Medical School, the University Hospital of Mainz, the University Clinic Schleswig-Holstein, Campus Kiel, the University Hospital Freiburg and the Charité University Hospital Berlin.

Norwegian patients in UC panel 1 were recruited through a population-based incidence study, the Inflammatory Bowel disease in South-Eastern Norway (IBSEN) study [1]. Dutch patients in UC panel 2 were recruited from the Academic Medical Center, Amsterdam and the University Medical Center, Groningen. German cases in UC panel 3 were recruited either at the Department of General Internal Medicine of the University Clinic Schleswig-Holstein (UKSH, Campus Kiel), the Charité University Hospital Berlin, through local outpatient services, or nationwide through the German IBD network of excellence (BMBF) or with the support of the German Crohn and Colitis Foundation (DCCV). British UC patients in UC panel 4 were recruited as described previously [2].

The Norwegian controls in PSC/UC panels 1 were randomly selected from the Norwegian Bone Marrow Donor Registry (NORDONOR). The Belgian and Dutch controls for PSC/UC panel 2 were recruited from Leuven, Belgium and from Utrecht, the Netherlands. The German controls of PSC/UC panel 3 were received from blood donors through the Northern German biobank Popgen ([www.popgen.de](http://www.popgen.de/)). The British controls of UC panel 4 were obtained from the 1958 British Birth Cohort ([http://www.b58cgene.sgul.ac.uk](http://www.b58cgene.sgul.ac.uk/)).

Overlap with previous studies: 90% of the UC patients and 72% the controls overlap with the study by Franke *et al.* [3]. There is an almost 100% overlap between PSC panel 1 and the Scandinavian patients genotyped for rs12612347 in Karlsen *et al*. [4] while PSC patients from Belgium, Netherlands and Germany were not genotyped for this SNP in that study.

*CLONING AND MUTAGENESIS OF HUMAN TGR5*

TGR5 was cloned using human liver cDNA as template and a forward primer with an *EcoRI* restriction-site (5'- CGGAATTCGCACTTGGTCCTTGTGCTCT-3') and a reverse primer with a *XhoI*-site (5'- GTCTCGAGTTAGTTCAAGTCCAGGTCGA-3'). The PCR product was cloned into the pcDNA3.1+ vector (Invitrogen, Karlsruhe, Germany). Human TGR5-yellow fluorescent protein (TGR5-YFP) was cloned into the pEYFP-N1 vector (Clontech, Palo Alto, CA, USA) as described [5]. A FLAG-TGR5-YFP plasmid having an N-terminal FLAG-tag (DYKDDDDK) and a C-terminal YFP-tag was cloned using TGR5-YFP as template and the following primers: hTGR5-FLAG-forward: 5'‑CGGAATTCCCGCCATGGACTACAAGGACGATGACGATAAGATGCACTTGGTCCTTGTGCTCT‑3'; hTGR5-FLAG-reverse: 5'‑CGGGATCCGCGTAGTTCAAGTCCAGGTCGA‑3'. The PCR product was cloned into the pEYFP-N1 vector. The sequences of the resulting plasmids TGR5, TGR5-YFP and FLAG-TGR5-YFP were confirmed by sequencing. Mutations were introduced into the *TGR5*-constructs using the Quickchange Multisite-mutagenesis kit (Stratagene, La Jolla, CA, USA) and the following primers:

W83R: 5'‑ CCGGGGTTACCGGTCCTGCCTCCTCG‑3',
A153V: 5'‑TGGACCCCTGGTGTCAACTGCAGCTCC‑3',
V178M: 5'‑TGCTGCCCGCCATGGGTGCTGCTGC‑3',
A217P: 5'‑CCGCCCTGGCCCGCCCCCTTACCTGGAGGC‑3',
S272G: 5'‑TAGGAAGTGCCGGTGCAGCGGCAGTG‑3',
Q296X: 5'‑GGGCAGCCGCCTAAAGGTGCCTGCAG‑3'.

Successful mutagenesis was verified by sequencing of the whole construct and alignment with the reference sequence (accession number [NM_001077191](http://www.ncbi.nlm.nih.gov/nuccore/NM_001077191?ordinalpos=1&itool=EntrezSystem2.PEntrez.Sequence.Sequence_ResultsPanel.Sequence_RVDocSum)).

# **REFERENCES**

1. Moum B, Vatn MH, Ekbom A, Aadland E, Fausa O, et al. (1996) Incidence of ulcerative colitis and indeterminate colitis in four counties of southeastern Norway, 1990-93. A prospective population-based study. The Inflammatory Bowel South-Eastern Norway (IBSEN) Study Group of Gastroenterologists. Scand J Gastroenterol 31: 362-366.

2. Onnie CM, Fisher SA, Pattni R, Sanderson J, Forbes A, et al. (2006) Associations of allelic variants of the multidrug resistance gene (ABCB1 or MDR1) and inflammatory bowel disease and their effects on disease behavior: a case-control and meta-analysis study. Inflamm Bowel Dis 12: 263-271.

3. Franke A, Balschun T, Karlsen TH, Sventoraityte J, Nikolaus S, et al. (2008) Sequence variants in IL10, ARPC2 and multiple other loci contribute to ulcerative colitis susceptibility. Nat Genet 40: 1319-1323.

4. Karlsen TH, Franke A, Melum E, Kaser A, Hov JR, et al. (2010) Genome-wide association analysis in primary sclerosing cholangitis. Gastroenterology 138: 1102-1111.

5. Keitel V, Cupisti K, Ullmer C, Knoefel WT, Kubitz R, et al. (2009) The membrane-bound bile acid receptor TGR5 is localized in the epithelium of human gallbladders. Hepatology 50: 861-870.

6. Venter JC, Adams MD, Myers EW, Li PW, Mural RJ, et al. (2001) The sequence of the human genome. Science 291: 1304-1351.
