# Supplementary material for: Mutational Characterization of the Bile Acid Receptor TGR5 in Primary Sclerosing Cholangitis
Source: PLoS One. 2010 Aug 25;5(8):e12403. doi: 10.1371/journal.pone.0012403 (PMC2928275; doi:10.1371/journal.pone.0012403)
Supplement: Results S1 — Supporting results from the resequencing of TGR5, the sequence collection, the structure modeling and the genetic associations at chromosome 2q35. (0.09 MB DOC) [file pone.0012403.s002.doc]

# **SUPPLEMENTARY RESULTS**

*RESEQUENCING OF TGR5* An important reference sequence error was noted in the coding part of *TGR5* exon 2, at basepair position 218,835,899 on chromosome 2, where the reference lacks one C base. All sequenced individuals carried CTGGCATTGCC**C**ACATTGCCAGGG instead of CTGGCATTGCCACATTGCCAGGG as presented in build 36 of the reference genome. The point is further supported by a sequence identical to our findings in the Celera genome [1], and has been corrected in NCBI build 37.

*SEQUENCE COLLECTION* Homologs of human *TGR5* were extracted from publicly available databases. *TGR5* homologs with the full protein-coding segment contained in a single exon, as in human *TGR5*, were detected in all mammalian genomes that have been sequenced with high coverage. Orthologs were also detected in the Carolina anole (a lizard), in two species of frog (*Xenopus Tropicalis* and *Xenopus Laevis*) and in multiple species of fish. No close orthologs were detected in data from chicken or zebra finch, in the primitive vertebrate *Petromyzon marinus* (sea lamprey) or in invertebrates including *Branchiostoma floridae* (lancelet) and the sea squirts *Ciona intestinalis* and *C. savignyi*. *TGR5* thus appears to be limited to vertebrates and might have been lost in birds.

Sequence identity between TGR5 orthologs is higher than 78% within placental mammals, ~70% between opossum and other mammals, and less than ~35% between mammals on the one hand and fish, frogs and lizard on the other. This might indicate that mammalian TGR5 proteins have a distinct function from those in other vertebrates. All mammalian TGR5 homologs are very likely to be bile acid receptors, but this may not be the case for the non-mammalian homologs.

A sequence alignment of TGR5 orthologs from 16 placental mammals, the marsupial opossum, lizard, two frog and four fish species is shown in Figures S2 and S3. The residues found to be mutated in PSC patients and healthy controls in the present study are conserved: Ser272 100% conserved in all vertebrates, Trp83 and Val178 in all mammals, and Ala153 and Ala217 in 16 out of 17 mammals. The introduction of a stop codon in Q296X deletes the C-terminal segment of TGR5 including a Ser-rich motif conserved in placental mammals.

*STRUCTURE MODELING* A DISOPRED2 [2] structural disorder prediction for human TGR5 gives a very strong signal for structural disorder in intracellular loop three (ICL3) (residues 197-221) and at the C-terminus (residues 300-330). The N-terminal segment is rich in proline and glycine residues (3 glycines and 4 pro in 19 N-terminal residues), which is a common signature of structural disorder. The remaining segments of the receptor appear to be structurally ordered and properly folded. As for the majority of G-protein-coupled receptors (GPCRs), the structure of TGR5 has not yet been determined experimentally. Currently, among several hundred human GPCRs [3], the experimental structure is only available for adenosine A2a receptor (hADORA2A), *i.e.* in Protein Data Bank (PDB) structure 3EML,[4] and beta-2 adrenergic receptor (hADRB2 , PDB identifier 3D4S) [5]. In addition, the structure has been solved for common turkey beta-1 adrenergic receptor (tADRB1, PDB id 2VT4)[6] and bovine rhodopsin (bRHO, PDB id 1F88) [7].

Sequences of human, mouse, dog, opossum, frog, anole, medaka, and fugu TGR5, hADORA2A, tADRB1, hADRB2, bRHO, and 38 additional human GPCRs were assembled and aligned with Muscle [8], TCoffee [9], MCoffee [10], and Expresso [11]. Based on these multiple sequence alignments (MSAs) and structural alignment of the GPCR structures, an MSA of the four possible templates and the human TGR5 target was generated. A detailed analysis of the experimental structures and the MSAs suggested hADORA2A (3EML) as the most promising template. The following sequence alignment of 3EML and human TGR5;

hTGR5 SPIPKGALGLSLALASLIITANLLLALGIAWDRRLRSPPAGCFFLSLLLAGLLTGLALPT

Template 3EML IMGSSVYITVELAIAVLAILGNVLVCWAVWLNSNLQ-NVTNYFVVSLAAADIAVGVLAIP

hTGR5 LPGLWNQS-RRGYWSCLLVYLAPNFSFLSLLANLLLVHGERYMAVLRPLQ-----PPGSI

Template 3EML FAITISTGFCAACHGCLFIACFVLVLTQSSIFSLLAIAIDRYIAIRIPLRYNGLVTGTRA

hTGR5 RLALLLTWAGPLLFASLPALGWNHWT-------PGANCSSQAIF-PAPYLYLEVYGLLLP

Template 3EML KGIIAICWVLSFAIGLTPMLGWNNCGQSQGCGEGQVACLFEDVVPMNYMVYFNFFACVLV

hTGR5 AVGAAAFLSVRVLATAHRQL----------------------------------------

Template 3EML PLLLMLGVYLRIFLAARRQLNIFEMLRIDEGLRLKIYKDTEGYYTIGIGHLLTKSPSLNA

hTGR5 ------------------------------------------------------------

Template 3EML AKSELDKAIGRNTNGVITKDEAEKLFNQDVDAAVRGILRNAKLKPVYDSLDAVRRAALIN

hTGR5 ------------------------------------------------------------

Template 3EML MVFQMGETGVAGFTNSLRMLQQKRWDEAAVNLAKSRWYNQTPNRAKRVITTFRTGTWDAY

hTGR5 -----LTWRQARAQAGAMLLFGLCWGPYVATLLLSVLAYEQRPPLGPGTLLSLLSLGSAS

Template 3EML RSTLQKEVHAAKSLAIIVGLFALCWLPLHIINCFTFFCPD-CSHAPLWLMYLAIVLSHTN

hTGR5 AAAVPVAMGLGDQRYTAPWRAAAQRCLQGL

Template 3EML SVVNPFIYAYRIREFRQTFRKIIRSHVLRQ

was submitted to the SwissModel homology-modeling server [12]. The resulting model was checked with standard analysis tools and found to be without obvious errors.

The structural model of human TGR5 is shown in Fig. 2 of the main manuscript. It is built from seven transmembrane helices (TMH1-7), three extracellular loops (ECL1-3), and three intracellular loops (ICL1-3). The expected accuracy of the model is as follows,

- Residues 1-10 (N-terminus), 197-217 (ICL3), and 303-330 (C-terminus) are in structurally disordered segments and are not included in the model.
- The transmembrane segments TMH1-7 are likely to be fairly correct, with all residues built into the correct TMH. Some of the detailed residue interactions are still likely to be incorrect.
- ICL1-2 and ECL1-3 have been modeled with low accuracy. The residues have been put in the correct ICL/ECL, but the geometry of the backbone and the residue side chains are most likely not correct. The disulfide bridge between the conserved residues Cys85 (ECL1) and Cys155 (ECL2) provides some constraints on the geometry of the ECLs.
- The accuracy of the model is sufficient to provide insight into the location and interactions of the residues found to be mutated in human PSC patients and controls.

*GENETIC ASSOCIATIONS AT CHROMOSOME 2q35
Details of the 2q35 fine-mapping pool including the analyses corrected for the rs11554825 SNP are shown below, p-values (<0.05) are marked in bold italic:*

| **Variant^A^** | **Position** | **Ctr MAF** | **Meta-analysis  PSC-UC** | | **PSC panel 1 subset**  **p-value** | **UC panel 3 subset**  **p-value** | **UC panel 4**  **p-value** | **Meta-analysis, corrected for TGR5 SNP rs11554825**  **p_rs11554825_** |
| --- | --- | --- | --- | --- | --- | --- | --- | --- |
|  |  |  | **OR (95% CI)** | **p-value** |  |  |  |  |
| rs10427230 | 218,661,520 | 0.05 | 1.24 (1.00, 1.52) | **0.045** | 0.98 | 0.16 | 0.073 | 0.14^LD1^ |
| rs17844669 | 218,697,261 | 0.48 | 0.84 (0.76, 0.93) | **0.00082** | 0.37 | **0.024** | **0.016** | 0.19 |
| rs4674259 | 218,699,250 | 0.48 | 0.84 (0.76, 0.93) | **0.0011** | 0.44 | **0.026** | **0.016** | 0.19 |
| rs6761387 | 218,704,798 | 0.04 | 1.00 (0.76, 1.31) | 0.99 | 0.33 | 0.38 | 0.12 | 0.70 |
| rs1567868 | 218,734,864 | 0.05 | 1.25 (1.02, 1.54) | **0.035** | 0.92 | 0.12 | 0.063 | 0.092 |
| rs1008563 | 218,735,133 | 0.43 | 1.12 (1.01, 1.24) | **0.027** | 0.52 | **0.042** | 0.35 | 0.87 |
| rs1008562 | 218,735,217 | 0.49 | 0.84 (0.76, 0.93) | **0.0011** | 0.51 | 0.0074 | **0.05** | 0.27 |
| rs16858784 | 218,735,242 | 0.04 | 0.95 (0.73, 1.23) | 0.70 | 0.31 | 0.13 | 0.86 | 0.95 |
| rs16858816 | 218,738,322 | 0.03 | 0.93 (0.69, 1.26) | 0.65 | 0.84 | 0.50 | 0.86 | 0.37 |
| rs3138060 | 218,739,745 | 0.05 | 1.30 (1.05, 1.60) | **0.014** | 0.92 | 0.085 | **0.021** | **0.049**^LD2^ |
| rs4674266 | 218,753,353 | 0.03 | 1.09 (0.83, 1.44) | 0.53 | 0.94 | 0.80 | 0.21 | 0.76 |
| rs4674269 | 218,759,036 | 0.05 | 1.33 (1.06, 1.66) | **0.011** | 0.74 | 0.46 | **0.00078** | **0.044**^LD3^ |
| rs12612347^B^ | 218,765,583 | 0.48 | 1.20 (1.09, 1.33) | **0.00037** | **0.014** | 0.10 | **0.019** | 0.55 |
| rs4674271 | 218,768,066 | 0.04 | 1.63 (1.29, 2.08) | **4.5x10^-5^** | 0.32 | **0.0028** | **0.0068** | **0.0025** |
| rs4674273 | 218,782,643 | 0.08 | 1.32 (1.10, 1.57) | **0.0025** | 0.22 | 0.059 | **0.039** | 0.059 |
| rs10932762 | 218,784,458 | 0.40 | 1.09 (0.98, 1.21) | 0.099 | 0.057 | 0.67 | 0.37 | 0.19 |
| rs13403276 | 218,789,542 | 0.07 | 0.95 (0.77, 1.17) | 0.63 | 0.51 | 0.71 | 0.94 | 0.65 |
| rs4674275 | 218,805,983 | 0.45 | 1.20 (1.08, 1.33) | **0.00043** | **0.039** | **0.047** | **0.031** | 0.41 |
| rs7605980 | 218,809,114 | 0.45 | 1.19 (1.08, 1.32) | **0.00068** | **0.033** | 0.054 | **0.049** | 0.47 |
| rs10169718 | 218,811,825 | 0.48 | 1.20 (1.09, 1.33) | **0.00041** | **0.021** | 0.057 | **0.037** | 0.70 |
| rs6436047 | 218,817,158 | 0.45 | 1.19 (1.07, 1.32) | **0.00082** | **0.037** | 0.055 | 0.054 | 0.48 |
| rs6436048 | 218,817,278 | 0.45 | 1.19 (1.08, 1.32) | **0.00080** | **0.033** | 0.057 | 0.055 | 0.53 |
| rs17462354 | 218,818,127 | 0.08 | 1.10 (0.91, 1.32) | 0.32 | 0.26 | 0.26 | 0.52 | 0.78 |
| rs13430006 | 218,821,334 | 0.48 | 1.19 (1.07, 1.31) | **0.00091** | **0.029** | 0.071 | 0.053 | 0.78 |
| rs3731859^C^ | 218,832,467 | 0.43 | 1.19 (1.08, 1.32) | **0.00067** | 0.075 | **0.017** | 0.088 | NA^¶^ |
| rs7571476 | 218,854,667 | 0.03 | 1.42 (1.06, 1.90) | **0.019** | 0.48 | **0.0014** | 0.65 | 0.18^LD4^ |
| rs1017697 | 218,878,706 | 0.03 | 0.89 (0.64, 1.24) | 0.49 | 0.50 | 0.876 | 0.17 | 0.17 |
| rs1017698 | 218,878,769 | 0.40 | 1.21 (1.09, 1.34) | **0.00026** | **0.021** | **0.016** | 0.088 | 0.49 |
| rs2014615 | 218,879,435 | 0.40 | 1.20 (1.08, 1.33) | **0.00050** | **0.030** | **0.018** | 0.12 | 0.54 |
| rs10932774 | 218,899,813 | 0.37 | 1.19 (1.07, 1.32) | **0.0010** | 0.064 | **0.0076** | 0.26 | 0.59 |
| rs11554825^E^ | 218,834,054 | 0.43 | 1.23 (1.11, 1.36) | **5.5x10^-5^** | **0.048** | **0.0024** | 0.059 | NA |

# ^A^Sixteen SNPs excluded; bad clustering in SNPlex genotyping: rs6711781, rs16858894, rs33990367. Deviation from Hardy-Weinberg-equilibrium (p<0.01) in controls: rs36094489, rs992157. Minor allele frequency<0.01 in cases or controls: rs6730826, rs34747313, rs17844701, rs2271541, rs10203642, rs10190292, rs5838686, rs10932773. Genotyping success rate<0.95: rs3092968, rs4791, rs4674282 ^B^Not part of the SNPlex fine-mapping pool, but data available in the context of other studies. ^C^rs3731859 is a surrogate for rs11554825 (*r*^2^=0.99). ^D^Strong linkage disequilibrium with rs11554825 (r2=0.99). ^E^*TGR5* SNP rs11554825 added after the sequencing experiment. ^LD1^*r*^2^=0.46 with rs4674271. ^LD2^*r*^2^=0.48 with rs4674271. ^LD3^*r*^2^=0.39 with rs4674271. ^LD4^*r*^2^=0.75 with rs4674271. CI: confidence interval; Ctr MAF: Overall minor allele frequency in the merged control panels of the meta-analysis; NA: not applicable; OR: odds ratio; SNP: Single-nucleotide polymorphism.

# **REFERENCES**

1. Ward JJ, Sodhi JS, McGuffin LJ, Buxton BF, Jones DT (2004) Prediction and functional analysis of native disorder in proteins from the three kingdoms of life. J Mol Biol 337: 635-645.

2. Foord SM, Bonner TI, Neubig RR, Rosser EM, Pin JP, et al. (2005) International Union of Pharmacology. XLVI. G protein-coupled receptor list. Pharmacol Rev 57: 279-288.

3. Jaakola VP, Griffith MT, Hanson MA, Cherezov V, Chien EY, et al. (2008) The 2.6 angstrom crystal structure of a human A2A adenosine receptor bound to an antagonist. Science 322: 1211-1217.

4. Hanson MA, Cherezov V, Griffith MT, Roth CB, Jaakola VP, et al. (2008) A specific cholesterol binding site is established by the 2.8 A structure of the human beta2-adrenergic receptor. Structure 16: 897-905.

5. Warne T, Serrano-Vega MJ, Baker JG, Moukhametzianov R, Edwards PC, et al. (2008) Structure of a beta1-adrenergic G-protein-coupled receptor. Nature 454: 486-491.

6. Palczewski K, Kumasaka T, Hori T, Behnke CA, Motoshima H, et al. (2000) Crystal structure of rhodopsin: A G protein-coupled receptor. Science 289: 739-745.

7. Edgar RC (2004) MUSCLE: multiple sequence alignment with high accuracy and high throughput. Nucleic Acids Res 32: 1792-1797.

8. Poirot O, O'Toole E, Notredame C (2003) Tcoffee@igs: A web server for computing, evaluating and combining multiple sequence alignments. Nucleic Acids Res 31: 3503-3506.

9. Moretti S, Armougom F, Wallace IM, Higgins DG, Jongeneel CV, et al. (2007) The M-Coffee web server: a meta-method for computing multiple sequence alignments by combining alternative alignment methods. Nucleic Acids Res 35: W645-648.

10. Armougom F, Moretti S, Poirot O, Audic S, Dumas P, et al. (2006) Expresso: automatic incorporation of structural information in multiple sequence alignments using 3D-Coffee. Nucleic Acids Res 34: W604-608.

11. Arnold K, Bordoli L, Kopp J, Schwede T (2006) The SWISS-MODEL workspace: a web-based environment for protein structure homology modelling. Bioinformatics 22: 195-201.
