# Supplementary material for: Mutational Characterization of the Bile Acid Receptor TGR5 in Primary Sclerosing Cholangitis
Source: PLoS One. 2010 Aug 25;5(8):e12403. doi: 10.1371/journal.pone.0012403 (PMC2928275; doi:10.1371/journal.pone.0012403)
Supplement: Figure S1 — Chromatograms from one heterozygote of each SNP detected through resequencing of TGR5. (1.14 MB DOC) [file pone.0012403.s003.doc]

| **SNP** | **One heterozygote sample, forward (left) and reverse (right) sequencing** |
| --- | --- |
| rs3731859 |  |
| TGR5snp1 |  |
| TGR5snp2 |  |
| rs13003334 |  |
| TGR5snp3 |  |
| rs11554825 |  |
| TGR5snp4 |  |
| TGR5snp5 |  |
| TGR5snp7 |  |
| TGR5snp6 |  |
| rs1567869 |  |
| TGR5snp8 |  |
| TGR5snp9 |  |
| TGR5snp10 |  |
| TGR5snp11 |  |
| rs57621524 |  |
| rs56192869 |  |
| TGR5snp12 |  |
| TGR5snp21,  W83R |  |
| TGR5snp22,  A153V |  |
| TGR5snp13,  V178M |  |
| TGR5snp14 |  |
| TGR5snp15 |  |
| TGR5snp16,  A217P^A^ |  |
| TGR5snp17, S272G |  |
| TGR5snp18,  Q296stop |  |
| TGR5snp19 |  |
| rs2292549 |  |
| TGR5snp20 |  |

^A^All individuals with this G>C substitution (TGR5snp16) did also have a (synonymous) G>C substitution in the position in front of it (TGR5snp15). The TGR5snp15 was however observed in several individuals without TGR5snp16.
