# Supplementary figures and images for: Mutational Characterization of the Bile Acid Receptor TGR5 in Primary Sclerosing Cholangitis
Source: PLoS One. 2010 Aug 25;5(8):e12403. doi: 10.1371/journal.pone.0012403 (PMC2928275; doi:10.1371/journal.pone.0012403)

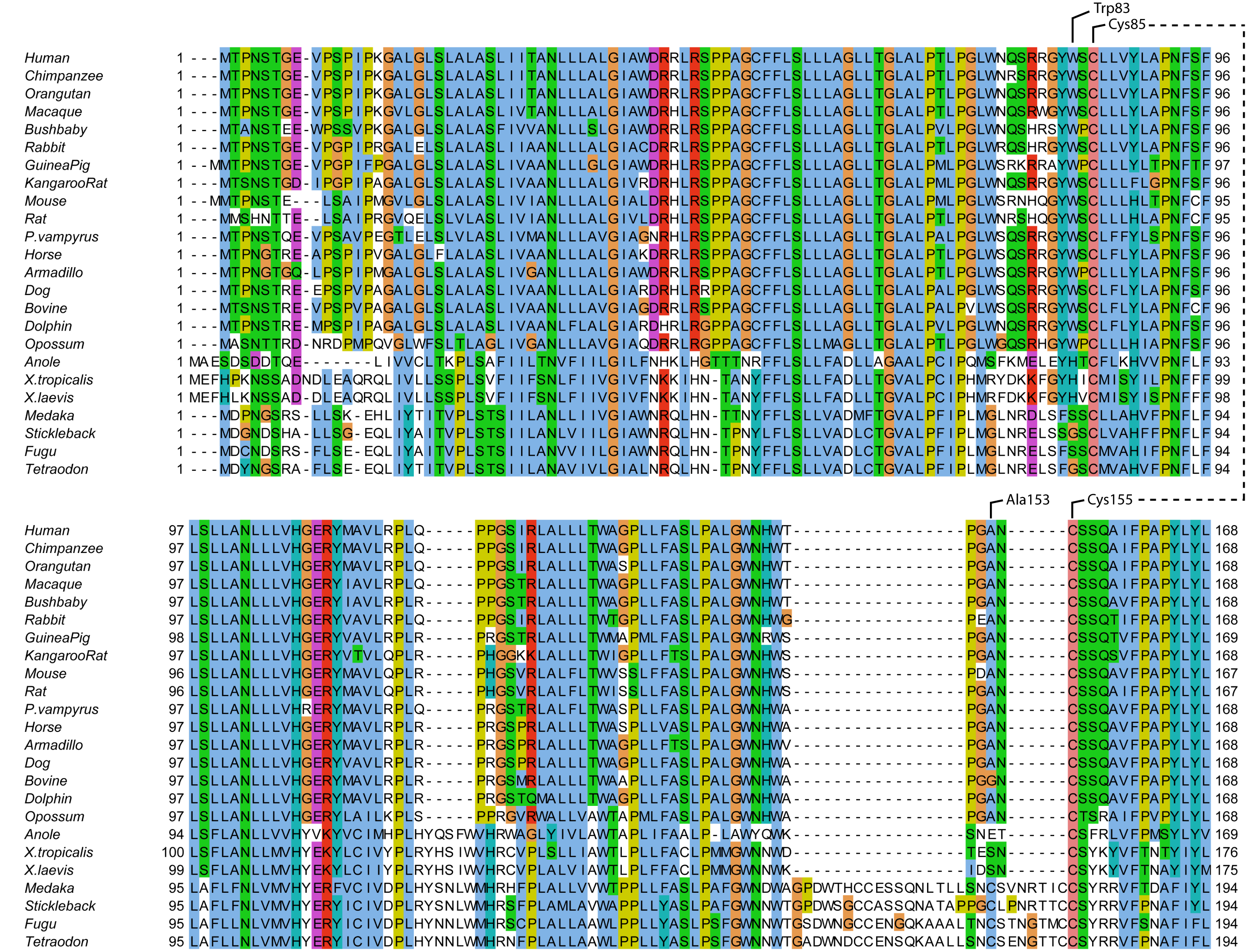

Supplement: Figure S2 — Multiple sequence alignment of human TGR5 and orthologs (N-terminal half). Human TGR5 and orthologs from the primates Pan troglodytes (chimpanzee), Pongo pygmaeus (orangutan), Macaca mulatta (macaque), and Otolemur garnettii (bushbaby), other mammals such as Oryctolagus cuniculus (rabbit), Cavia porcellus (guinea pig), Dipodomys ordii (kangaroo rat), Mus musculus (mouse), Rattus norvegicus (rat), Pteropus vampyrus (flying fox bat), Equus caballus (horse), Dasypus novemcinctus (armadillo), Canis familiaris (dog), Bos taurus (cow), Tursiops truncatus (dolphin), the marsupial Monodelphis domestica (opossum), the lizard Anolis carolinensis, the frogs Xenopus tropicalis and X. laevis, and the fish Oryzias latipes (medaka), Gasterosteus aculeatus (three-spined stickleback), Takifugu rubripes (fugu), and Tetraodon nigroviridis. Residues found to be mutated in humans have been highlighted, and the Cys85-Cys155 disulfide bridge has been indicated (dashed line). (3.55 MB TIF) [file pone.0012403.s004.tif]

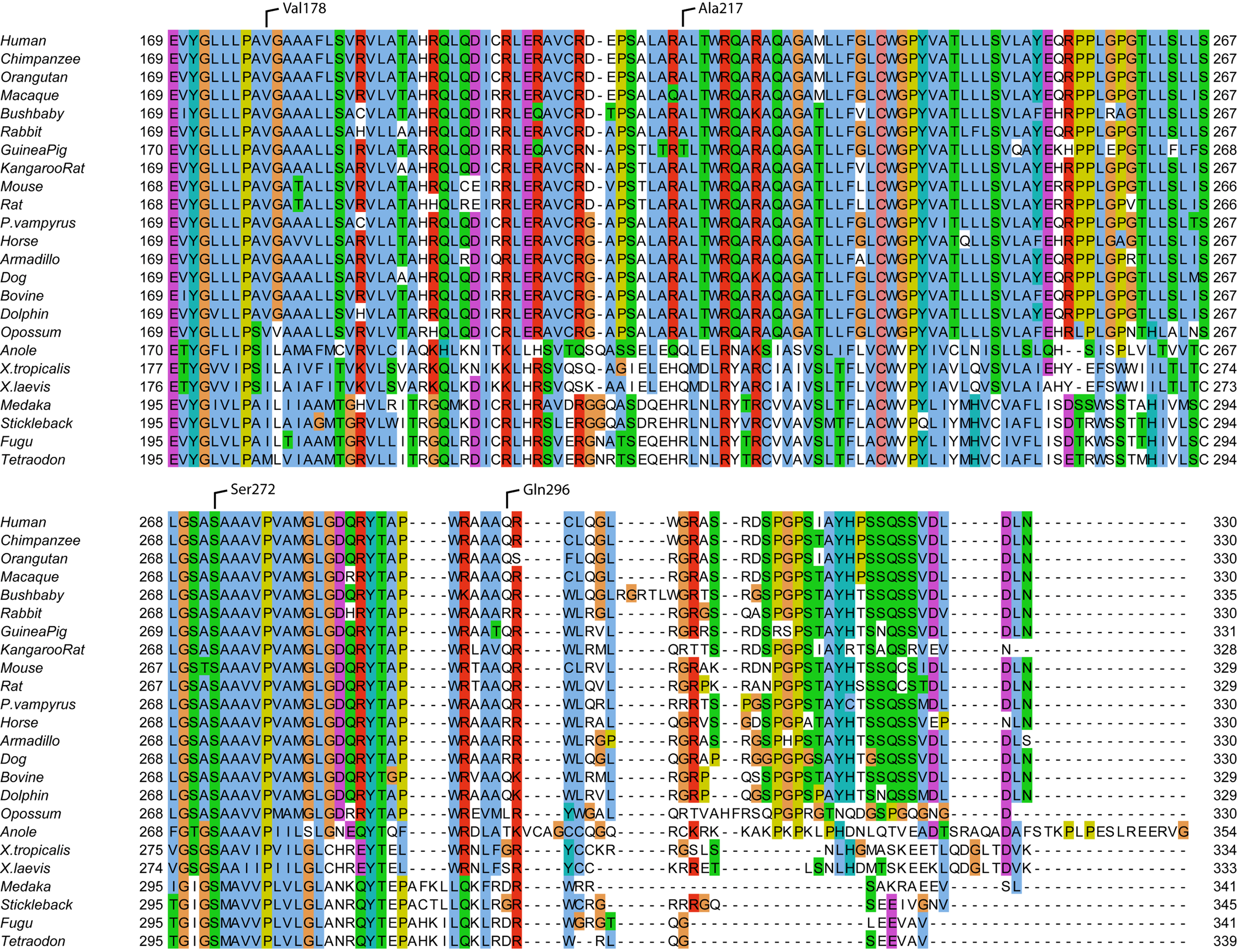

Supplement: Figure S3 — Multiple sequence alignment of TGR5 (C-terminal half) from the same species as in Figure S2. Residues found to be mutated in humans have been highlighted. (6.29 MB TIF) [file pone.0012403.s005.tif]

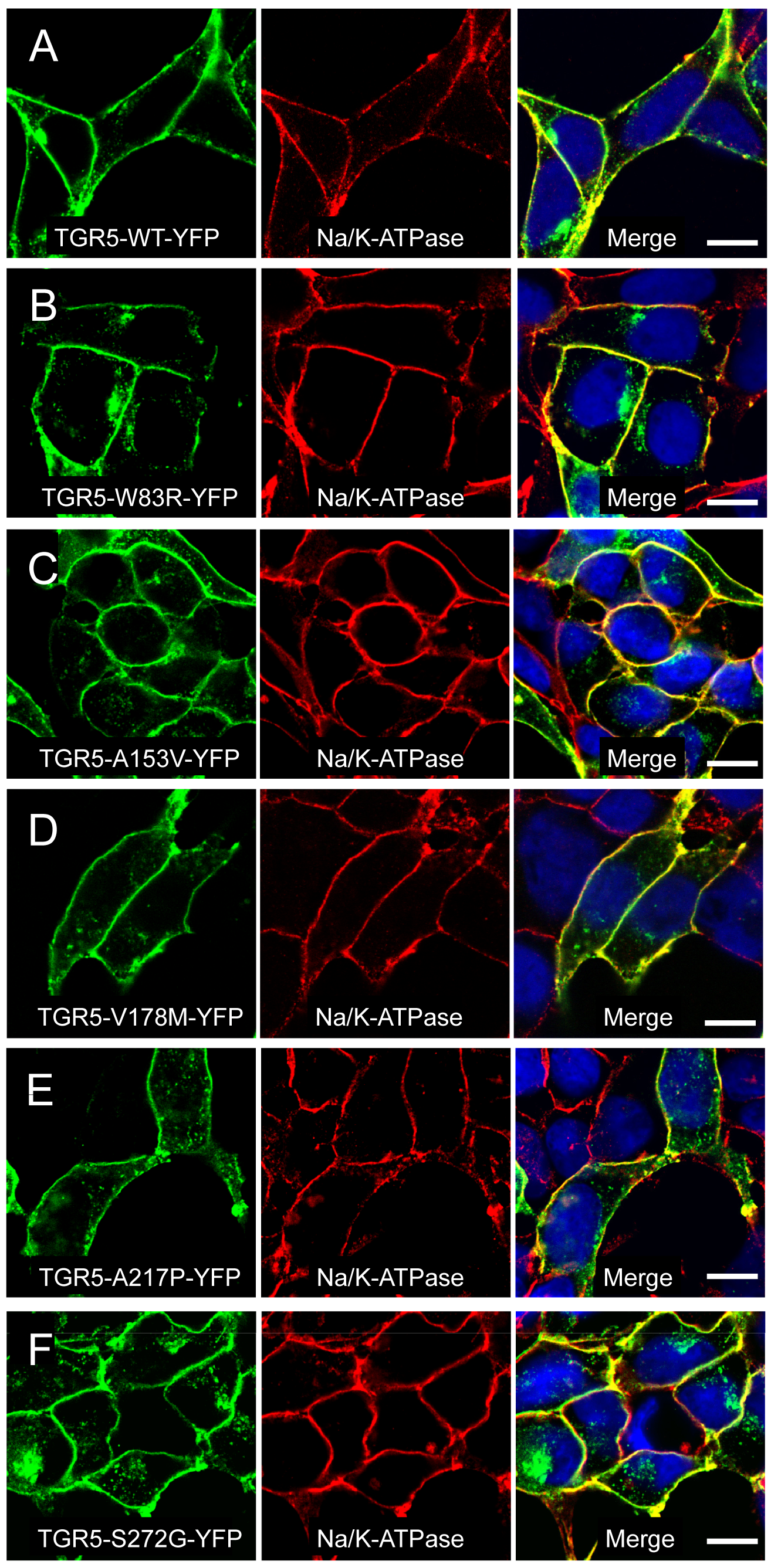

Supplement: Figure S4 — Localization of TGR5-YFP variants in HEK293 cells. HEK293 cells were transiently transfected with the different TGR5-YFP variants. An antibody against Na+/K+-ATPase was used to stain the plasma membrane (shown in red). Nuclei were stained with Hoechst (blue). A. Wildtype TGR5 (WT) was almost completely targeted to the plasma membrane. B. The mutant TGR5-W83R-YFP was also localized in the plasma membrane as demonstrated by the colocalization with the Na+/K+-ATPase resulting in a yellow coloring in the overlay picture. C. Introduction of A153V into TGR5-YFP did not affect plasma membrane localization. D. TGR5-V178M-YFP was also targeted to the plasma membrane as shown by the colocalization with the Na+/K+-ATPase fluorescence pattern. E. TGR5-A217P-YFP was also detected in the plasma membrane as demonstrated by the yellow coloring in the superimposed image. F. TGR5-S272G-YFP was both localized in the plasma membrane but also in some intracellular vesicles. Bars = 10 µm. (5.32 MB TIF) [file pone.0012403.s006.tif]

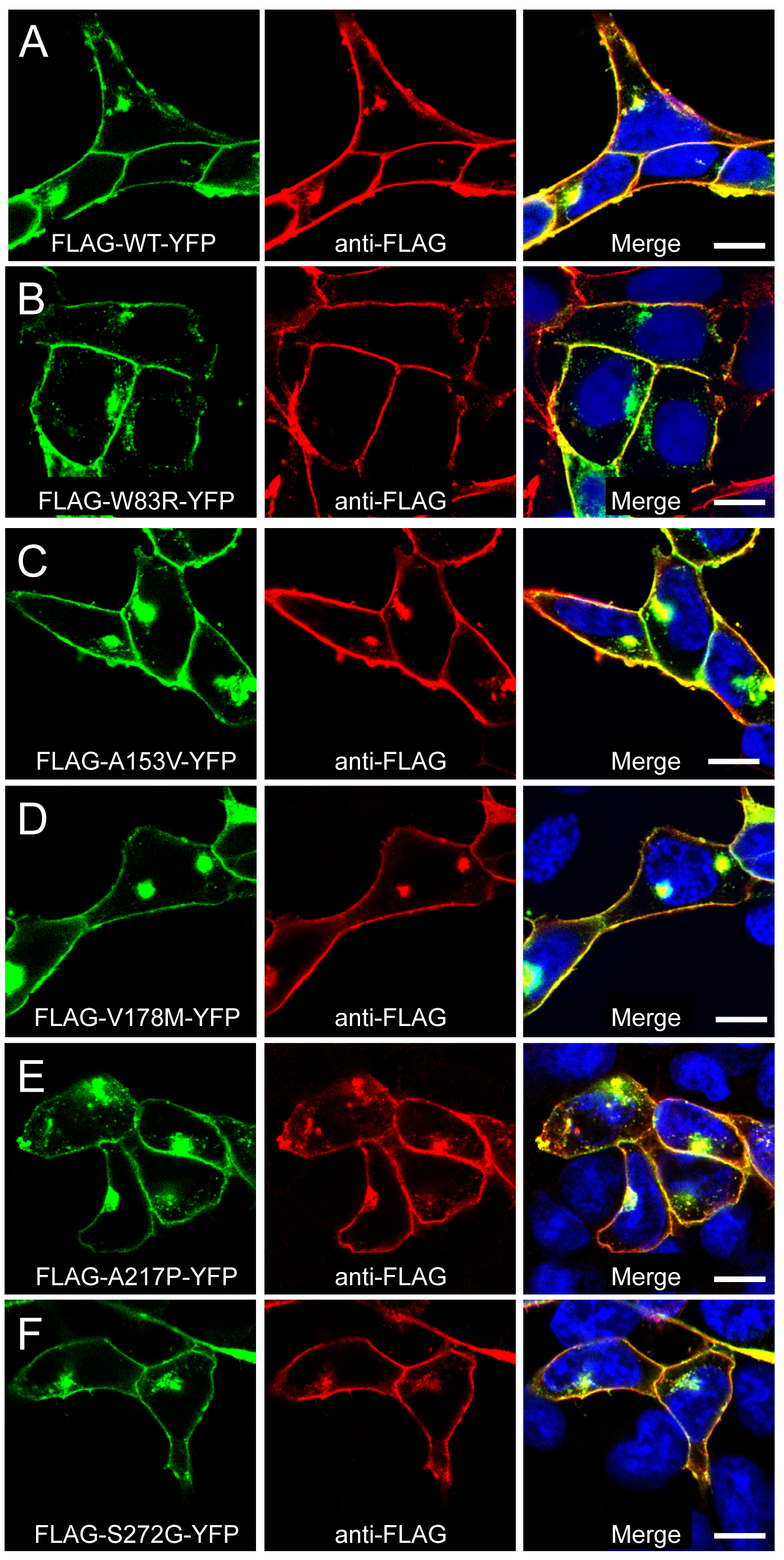

Supplement: Figure S5 — Localization of FLAG-TGR5-YFP in HEK293 cells. HEK293 cells were transiently transfected with the different FLAG-TGR5-YFP constructs. The FLAG-tag was made visible using an anti-FLAG-M2 antibody (in red). The yellow coloring in the overlay images demonstrate that the FLAG antibody completely binds to the FLAG-TGR5-YFP proteins both in the plasma membrane as well as in intracellular vesicles (A–F). Nuclei were stained with Hoechst. Bars = 10 µm. (4.35 MB TIF) [file pone.0012403.s007.tif]

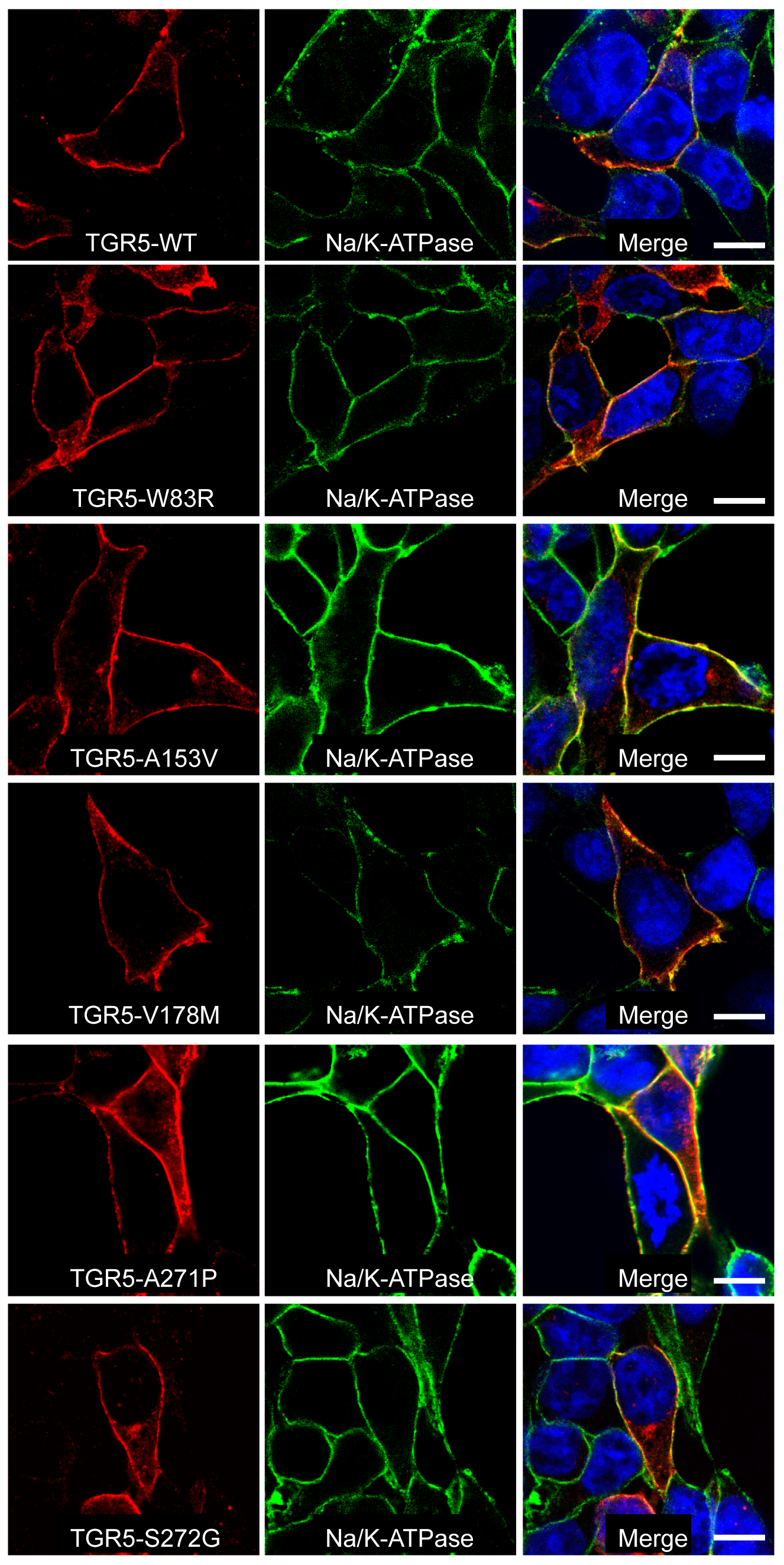

Supplement: Figure S6 — Localization of TGR5 in pcDNA in HEK293 cells. HEK293 cells were transiently transfected with the different TGR5 variants (without tags) and stained for TGR5 using the anti-TGR5 (M39) antibody (shown in red). An antibody against Na+/K+-ATPase was used as a marker for the plasma membrane (shown in green). Nuclei were stained with Hoechst (blue). Wildtype TGR5 (TGR5-WT) was almost completely targeted to the plasma membrane. All TGR5-variants were also localized in the plasma membrane of HEK293 cells as demonstrated by the colocalization with the fluorescence from the Na+/K+-ATPase antibody. (4.21 MB TIF) [file pone.0012403.s008.tif]

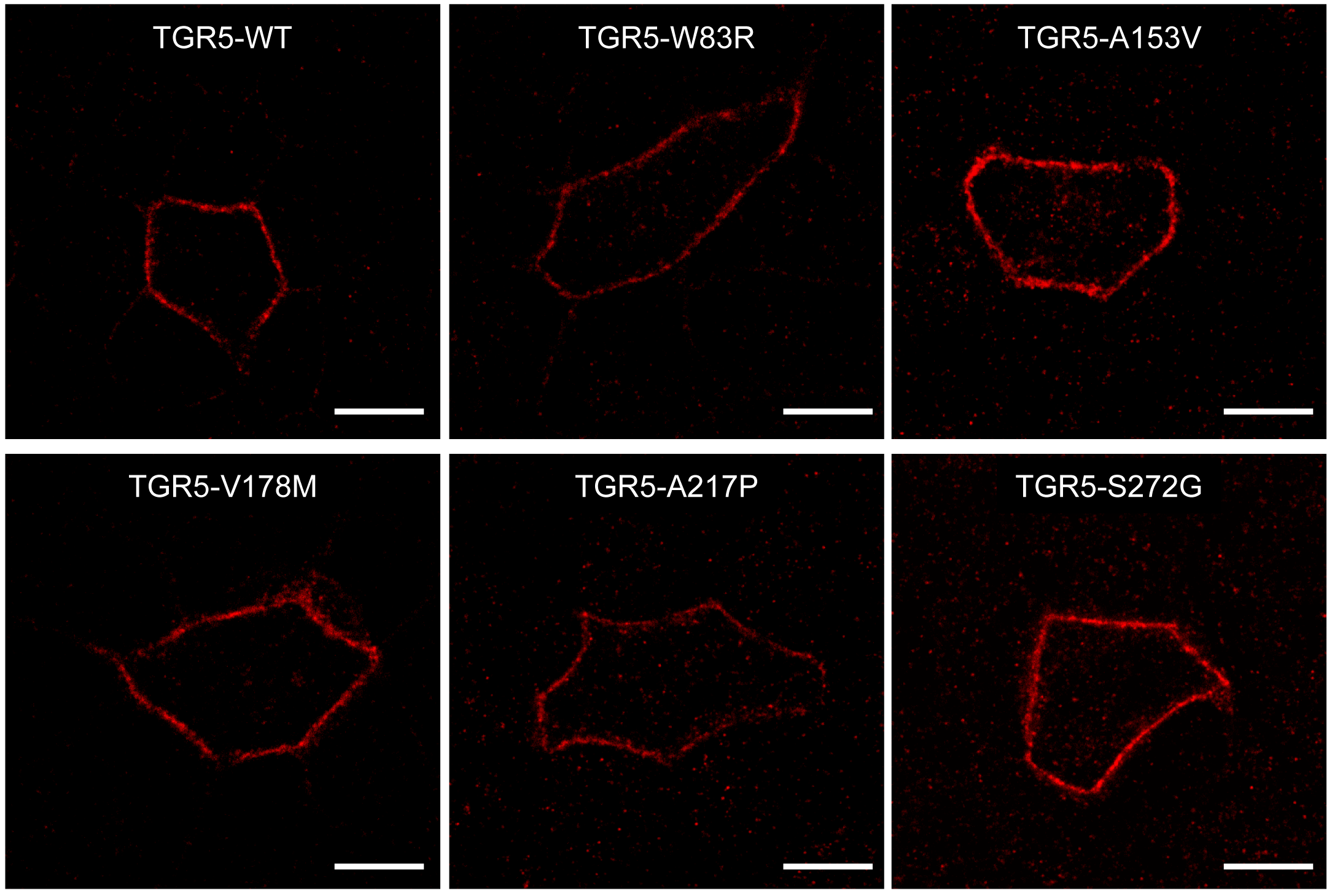

Supplement: Figure S7 — Localization of TGR5 in pcDNA in MDCK cells. TGR5 variants were transfected into polarized MDCK cells. TGR5 and TGR5 mutants were all detected in the plasma membrane using the anti-TGR5 antibody (M39 in red). Bars = 10 µm. (1.29 MB TIF) [file pone.0012403.s009.tif]

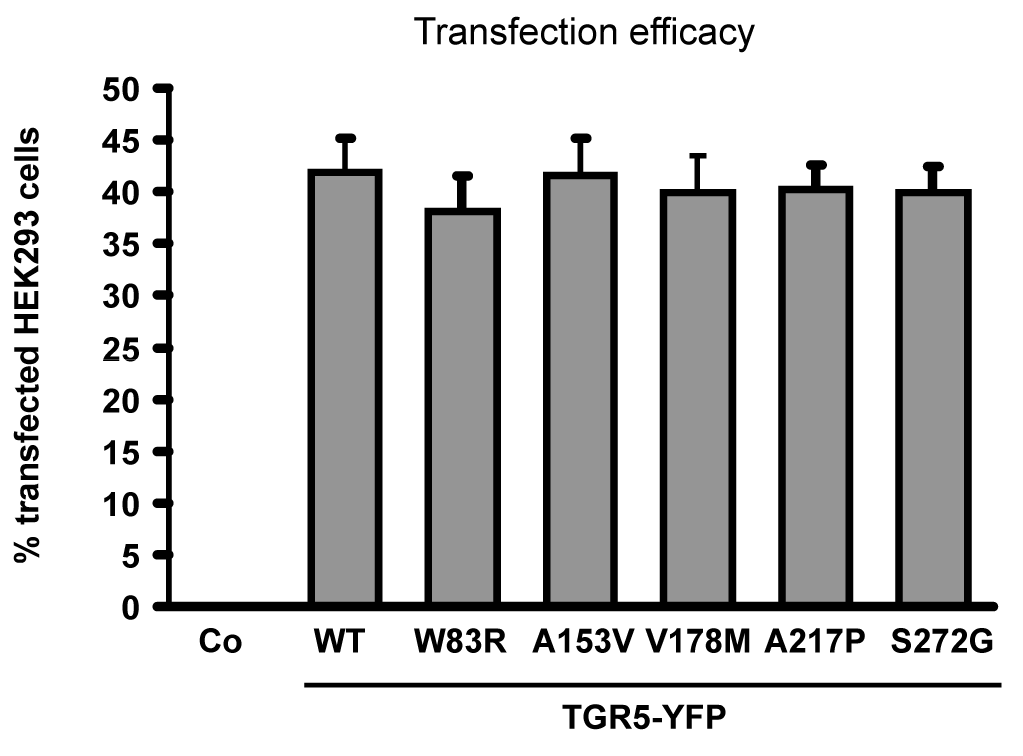

Supplement: Figure S8 — Transfection efficacy of TGR5-YFP in HEK293 cells. HEK293 cells were transiently transfected with TGR5-YFP. Transfection efficacy was determined by flow cytometry. (0.10 MB TIF) [file pone.0012403.s010.tif]

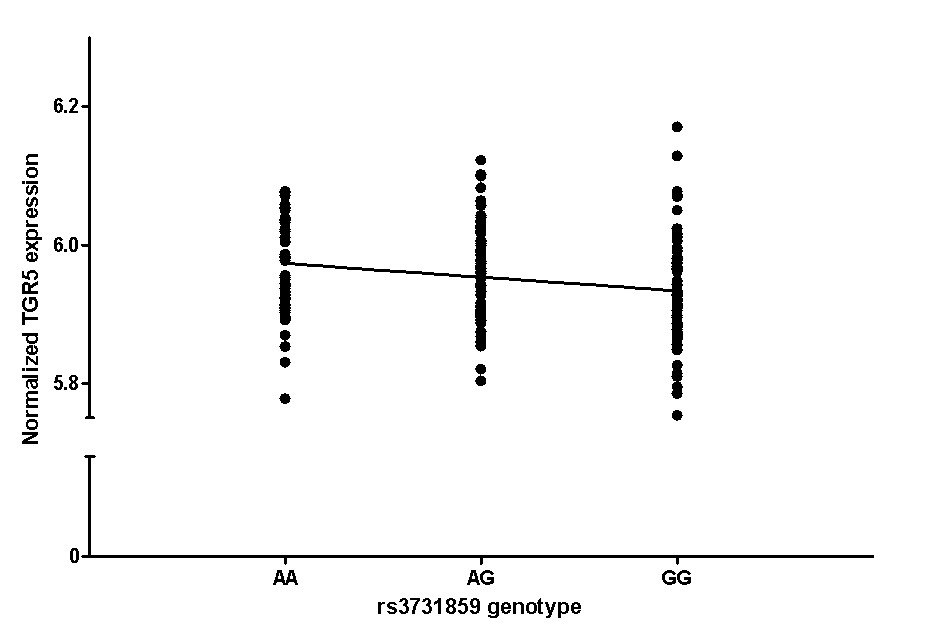

Supplement: Figure S9 — TGR5 expression in Epstein-Barr-virus-transformed lymphoblastoid cell lines according to rs3731859 genotypes in all non-related HapMap individuals (n = 210). Expression levels were retrieved from the GENEVAR project. Genotypes and height of expression were significantly correlated (r 2 = 0.048, p = 0.0015) in a linear regression analysis performed in SNPexp v1.1 (http://app3.titan.uio.no/biotools/tool.php?app=snpexp, which utilizes Plink v1.06). (0.04 MB TIF) [file pone.0012403.s011.tif]
