# Supplementary material for: Mutational Characterization of the Bile Acid Receptor TGR5 in Primary Sclerosing Cholangitis
Source: PLoS One. 2010 Aug 25;5(8):e12403. doi: 10.1371/journal.pone.0012403 (PMC2928275; doi:10.1371/journal.pone.0012403)
Supplement: Table S1 — Details of the nine primer pairs used in TGR5 resequencing (M13-primer-sequence underlined). (0.04 MB DOC) [file pone.0012403.s012.doc]

| **Amplicon name** | **Forward primer^A^** | **Reverse primer** | **Touchdown PCR: Tm [°C]** | **Amplicon size (bp)** |
| --- | --- | --- | --- | --- |
| 5’UTR amplicon 1 | tgtaaaacgacggccagtTATCAGAAGGGTCCCACACC | caggaaacagctatgaccACCTGGCCCCTCTAAGACAG | 69 | 639 |
| 5’UTR amplicon 2 | tgtaaaacgacggccagtAGAGCACCAGAGTCCCTTTG | caggaaacagctatgaccTGATGAAGAGGCATGAGCTG | 69 | 613 |
| 5’UTR amplicon 3 | tgtaaaacgacggccagtTCATTTGCCCAAGTTCACTG | caggaaacagctatgaccCACCCAGTTCCCCTCTCC | 69 | 661 |
| Exon 1 | tgtaaaacgacggccagtGAAGTCGGGAGGGGTAAGTC | caggaaacagctatgaccCAAACCTGGCCTGAGCTG | 69 | 655 |
| Intron | tgtaaaacgacggccagtGCTACTACACCCAGCCCAAG | caggaaacagctatgaccTCATCTGTCCCTGTCCTTCC | 69 | 640 |
| Exon 2 amplicon 1 | tgtaaaacgacggccagtAGCATCTTCCTTCCTCTCAGC | caggaaacagctatgaccTTGTGTATCCCTGCCTCCAC | 69 | 653 |
| Exon 2 amplicon 2 | tgtaaaacgacggccagtTGGAGCCTGTCACCTAATGG | caggaaacagctatgaccCCAGCAGTAGGCTCAGGAAG | 65 | 697 |
| Exon 2 amplicon 3 | tgtaaaacgacggccagtATCATCACCGCGAACCTG | caggaaacagctatgaccCATAGGCCAGGACTGAGAGG | 65 | 672 |
| Exon 2 amplicon 4 | tgtaaaacgacggccagtCTGCTGCCTTCCTCTCTGTC | caggaaacagctatgaccTCCACGCGTATGTCTGTAGG | 66 | 688 |
