# Supplementary material for: Mutational Characterization of the Bile Acid Receptor TGR5 in Primary Sclerosing Cholangitis
Source: PLoS One. 2010 Aug 25;5(8):e12403. doi: 10.1371/journal.pone.0012403 (PMC2928275; doi:10.1371/journal.pone.0012403)
Supplement: Note S1 — List of members in the IBSEN study group. (0.03 MB DOC) [file pone.0012403.s013.doc]

# **SUPPLEMENTARY NOTE**

*The following people belong to the the Inflammatory Bowel disease in South-Eastern Norway (IBSEN) study group:*

Morten H. Vatn, Oslo University Hospital Rikshospitalet, Clinic for Specialized Medicine and Surgery, 0027 Oslo, Norway and Epigen, Akershus University Hospital and the University of Oslo, 1474 Oslo, Norway

Bjørn Moum, Oslo University Hospital Aker, 0514 Oslo, Norway

Jørgen Jahnsen, Oslo University Hospital Aker, 0514 Oslo, Norway

Camilla Solberg, Oslo University Hospital Ullevål, 0407 Oslo, Norway

Njål Stray, Diakonhjemmets Hospital, 0319 Oslo, Norway

Magne Henriksen, Østfold Hospital, 1603 Frederikstad, Norway

Jostein Sauar, Telemark Hospital, 3710 Skien, Norway

Ole Høie, Sørlandet Hospital, 4809 Arendal, Norway
